# Non-coding RNAs: master regulators in the battle between host antiviral defense and viral replication

**DOI:** 10.3389/fimmu.2026.1908472

**Published:** 2026-09-14

**Authors:** Na Chen, Baoge Zhang, Xiaoyong Chen, Haiyan Zhang, Ziding Yu

**Affiliations:** 1 School of Food and Bioengineering, Wuhu Vocational Technical University, Wuhu, China; 2 Department of Pathogen Biology, School of Basic Medical Sciences, Fujian Medical University, Fuzhou, China; 3 Key Laboratory of Gastrointestinal Cancer (Fujian Medical University), Ministry Education, Fuzhou, China; 4 MOE Joint International Research Laboratory of Animal Health and Food Safety, Engineering Laboratory of Animal Immunity of Jiangsu Province, College of Veterinary Medicine, Nanjing Agricultural University, Nanjing, China; 5 Fujian Academy of Chinese Medical Sciences, Fuzhou, China; 6 Xingzhi College Zhejiang Normal University, Jinhua, China; 7 Detection of Food-borne Pathogenic Microorganisms Engineering Research Center of Wuhu, Wuhu, China

**Keywords:** antagonism, host antiviral immunity, molecular hijacking, non-coding RNA, signaling pathways, viral replication cycle, virus-host interactions

## Abstract

Viral infectious diseases pose serious threats to global public health. Host non-coding RNAs (ncRNAs), including miRNAs, lncRNAs, circRNAs, and vtRNAs, act as master regulators of host antiviral defense and viral replication. Current studies on ncRNA-virus interactions are generally fragmented, and most reviews focus on a single virus or a single class of ncRNAs, without combining ncRNA expression dynamics with their biological functions for systematic collation. Herein, we establish a novel four-category classification framework based on ncRNA expression patterns and functional characteristics upon viral infection. To counteract viral infections, the host dynamically modulates the expression of distinct ncRNAs to target proviral host factors, host restriction factors, other ncRNAs, micropeptides, and lipids, thereby orchestrating antiviral innate immune signaling pathways, viral replication cycles, autophagy, apoptosis, metabolic reprogramming, and stress granule formation to establish a multilayered antiviral defense system. Concurrently, representative viruses posing severe threats to global public health, including IAV, DENV, HCV, ZIKV, HIV-1, and SARS-CoV-2, have evolved sophisticated strategies to hijack host ncRNA networks and antagonize host antiviral defenses. This review systematically summarizes and elaborates the conserved regulatory principles of various ncRNAs in the host antiviral response, clarifies the molecular mechanisms by which viruses hijack and antagonize host ncRNAs, further refines the theoretical framework of host ncRNA-virus interactions centered on viral replication regulation, discusses the future research directions under this system, and thus provides a theoretical basis for the development of host ncRNA-based antiviral drugs.

## 1 Introduction

Viral infection poses a formidable global public health threat, with representative pathogenic viruses including influenza A virus (IAV), dengue virus (DENV), hepatitis C virus (HCV), Zika virus (ZIKV), human immunodeficiency virus type 1 (HIV-1), and severe acute respiratory syndrome coronavirus 2 (SARS-CoV-2) triggering severe infectious diseases (including regional outbreaks, chronic epidemics, and even global pandemics) through efficient replication, immune evasion, and/or cross-species transmission, inflicting severe impacts on human health and socioeconomic development (1–5). As the first line of defense against viral invasion, host innate immunity recognizes viral pathogen-associated molecular patterns via PRRs and activates downstream IFN signaling, ISGs expression, and other related signaling pathways, coordinately regulating cellular physiological processes such as autophagy, apoptosis, metabolic reprogramming, and stress granule formation to construct a multidimensional antiviral defense network (1, 6–8). However, in recent years, the discovery and functional characterization of ncRNAs have challenged the traditional understanding of gene regulation limited to protein-coding genes. As a class of RNA molecules with no protein-coding capacity but crucial biological functions, major ncRNA classes, including microRNAs (miRNAs), long non-coding RNAs (lncRNAs), circular RNAs (circRNAs), and vault RNAs (vtRNAs), have been identified as master regulators in the battle between host antiviral defense and viral replication (9). Specifically, miRNAs are ~22-nucleotide short ncRNAs, lncRNAs are >200-nucleotide RNAs with no protein-coding potential, circRNAs are ncRNA molecules lacking 5′ caps and 3′ poly(A) tails, and vtRNAs are 84-141-nt eukaryotic ncRNAs transcribed by RNA polymerase III. By exerting precise regulatory effects at multiple levels, including transcription, translation, post-translational modification, and epigenetics, ncRNAs act as a core molecular link connecting host innate immunity and viral replication, and the ncRNA-mediated virus-host interactions have become a cutting-edge research hotspot in the field of antiviral study (1). A growing body of high-impact studies has confirmed that ncRNAs represent highly promising antiviral drug targets, offering valuable directions for the development of novel targeted therapies (9–14).

Mounting research demonstrates abundant host ncRNAs regulate viral replication through direct or indirect modulation of every functional node within core RNA-sensing innate immune signaling cascades. Upon viral RNA invasion, host pattern recognition receptors RIG-I and MDA5 detect pathogen-derived RNA ligands and recruit the adaptor protein MAVS to propagate downstream immune signals; TLR3 and TLR7 rely on TRIF and MyD88 adaptor proteins for signal transduction, respectively. These upstream adaptor complexes recruit and phosphorylate TBK1 and IKK family kinases, which drive activation and nuclear translocation of transcription factors IRF3 and IRF7. Once translocated into the nucleus, these transcription factors bind ISRE and κB promoter motifs to trigger robust transcription and secretion of type I and III interferons. Secreted IFNs engage cell-surface IFNAR receptors to activate the JAK-TYK-STAT signaling cascade, facilitating assembly of the ISGF3 transcriptional complex. ISGF3 further drives broad-spectrum induction of ISGs including Mx, IFITM, PKR and Viperin, whose combined activities suppress multiple stages of viral transcription, translation and virion assembly to establish cell-intrinsic antiviral immunity. Every checkpoint along this RNA-sensing immune axis is precisely tuned by diverse ncRNA species, such as miRNAs, lncRNAs, circRNAs and vtRNAs, forming a multi-layered regulatory network that underpins all virus-host crosstalk detailed in the subsequent sections (1, 9).

Under the pressure of viral infection, the host has evolved a highly sophisticated ncRNA regulatory strategy to initiate antiviral defense, which is centered on the dynamic modulation of the expression levels of distinct ncRNAs to construct a multilayered and comprehensive antiviral regulatory network. On the one hand, viral infection can induce a significant upregulation of host antiviral ncRNAs, which block viral proliferation by directly targeting viral genomes, viral-encoded proteins, or key stages of the viral replication cycle (e.g., regulation of polymerase activity, nuclear transport, and aggregation of viral proteins). These ncRNAs can either directly and positively regulate innate immune signaling pathways (such as the pathways mediated by TLR3/TLR7 and RIG-I/MDA5) or target proviral host factors to relieve their suppression of immune signaling, thereby amplifying the IFN-ISGs antiviral response. Furthermore, they can also stabilize the expression of antiviral factors through mechanisms including ceRNA sponge, protein decoy effects, and regulation of histone modifications, or restrict viral replication by regulating key cellular physiological processes (15). On the other hand, the host can downregulate endogenous proviral ncRNAs to abrogate their inhibition on host restriction factors and negative regulation on innate immunity, thus weakening the proviral effects of these ncRNAs and enhancing host antiviral capacity (16). The regulatory functions of different ncRNA classes are not isolated but rather synergistic and complementary, participating in host antiviral defense in multiple dimensions from viral recognition and immune activation to the regulation of cellular physiological processes (apoptosis, metabolic reprogramming, stress granule formation, etc.) and forming a core molecular barrier for the host to cope with viral infection.

Conversely, to achieve efficient replication and immune evasion, pathogenic viruses have evolved complex and elaborate strategies to counteract host ncRNA-mediated antiviral defense during long-term co-evolution with the host, which are mainly manifested in two core modes: hijacking the host ncRNA network and antagonizing the antiviral functions of ncRNAs. Viruses can upregulate the expression of host proviral ncRNAs or directly utilize endogenous host ncRNAs to target and regulate autophagy, cellular apoptosis, and metabolic reprogramming. Meanwhile, they can convert host ncRNAs into “tools” for their own replication by promoting key stages of the viral replication cycle, inhibiting antiviral innate immune signaling pathways, regulating host restriction factors, and encoding functional micropeptides, thereby constructing a cellular microenvironment permissive for viral proliferation. Additionally, viruses can specifically downregulate host antiviral ncRNAs or interfere with their binding to target molecules, thereby blocking their functional exertion and abrogating their inhibition of viral gene expression and replication, thus antagonizing the host antiviral defense at the ncRNA level (17, 18). Notably, the regulation of the ncRNA network by different viruses exhibits both evolutionary conservation (e.g., miR-146a and miR-125a exert proviral effects in multiple viral infections) and species specificity (e.g., lncRNA-GAS5 exerts completely opposite regulatory functions in IAV and HCV infections) (19–26). For instance, avian influenza virus, a zoonotic IAV variant, has been identified as a classic model for studying the species-specific ncRNA regulation and cross-species transmission barriers of IAV, and its regulatory mechanisms of host ncRNAs also provide critical insights for understanding the adaptive evolution of human IAV strains in host cells. This characteristic of coexistent conservation and specificity reflects the complexity and diversity of the ncRNA regulatory game between viruses and hosts (27, 28).

Recently, the rapid development of high-throughput sequencing and molecular biology technologies has promoted the systematic characterization of the regulatory functions of ncRNAs in virus-host interactions, and the regulatory mechanisms of a series of master ncRNAs have been gradually elucidated, providing a novel perspective for deciphering the molecular pathogenic mechanisms of viral infection. Current research in this field mostly focuses on the functional properties (antiviral or proviral) and specific molecular pathways of host ncRNAs in regulating viral replication, yet does relatively neglect the expression regulatory characteristics of ncRNAs themselves after viral infection, and fails to perform a systematic correlation analysis between the functional properties of host ncRNAs and their expression changes (upregulation/downregulation) post infection (29–33). This separation of research perspectives not only results in insufficient attention to the host-specific antiviral defense strategy of “proviral ncRNAs downregulated after infection” but also makes it difficult to form a systematic understanding of the core regulatory logic of viruses hijacking and antagonizing host ncRNAs. Based on this, we propose a four-category classification framework combining the two dimensions to systematically sort out the regulatory rules of ncRNA-mediated virus-host interactions: first, ncRNAs exert antiviral effects and are upregulated after infection, representing a classic antiviral defense strategy of the host; second, ncRNAs promote viral replication and are downregulated after infection, being another easily overlooked antiviral defense strategy of the host; third, ncRNAs promote viral replication and are upregulated after infection, serving as the core mode for viruses to hijack host ncRNAs to meet their own proliferation needs; fourth, ncRNAs exert antiviral effects and are downregulated after infection, acting as a key strategy for viruses to antagonize host ncRNA-mediated antiviral defense. Based on this framework, this review systematically summarizes the regulatory principles of major ncRNAs, including miRNAs, lncRNAs, circRNAs, and vtRNAs in the host antiviral response, clarifies the molecular mechanisms by which major pathogenic viruses such as IAV, DENV, HCV, ZIKV, HIV-1, and SARS-CoV-2 hijack and antagonize host ncRNAs, further refines the theoretical framework of host ncRNA-virus interactions centered on viral replication regulation, sorts out the evolutionarily conserved regulatory principles of ncRNA-mediated virus-host interactions, and discusses the future research directions under this research system. It aims to provide a solid theoretical basis for the subsequent development of host ncRNA-based antiviral drugs and new ideas and strategies for addressing the global public health challenges caused by viral infection. To visualize this unified four-category organizational logic and facilitate readers’ rapid comprehension of the overall structure of this review, we provide a hierarchical schematic (**Figure 1**) illustrating the two core classification dimensions and four functional subgroups of ncRNA–host–virus crosstalk.

**Figure 1:**
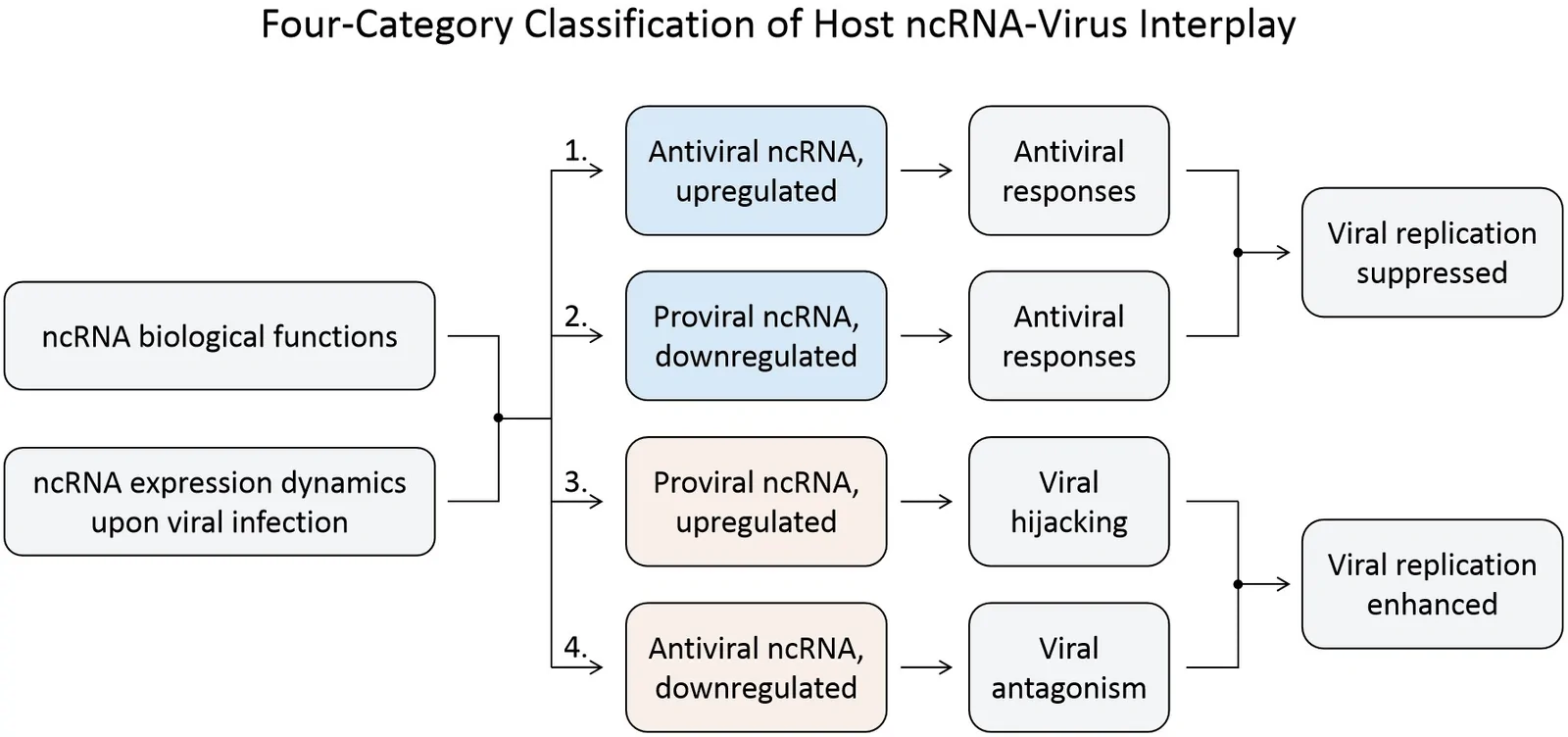
Four-category hierarchical framework of host ncRNA–virus interplay during viral infection. Host ncRNA–virus interactions are systematically categorized based on two intersecting dimensions: ncRNA biological functions and ncRNA expression dynamics upon viral infection. This schematic defines four distinct regulatory categories: (1) upregulated antiviral ncRNAs; (2) downregulated proviral ncRNAs; (3) upregulated proviral ncRNAs; (4) downregulated antiviral ncRNAs. Categories 1 and 2 collectively mediate host-initiated antiviral responses that suppress viral replication. By contrast, Category 3 represents viral hijacking of host ncRNA networks, and Category 4 denotes viral antagonism against host protective ncRNAs; both virus-driven regulatory modes enhance viral replication. This hierarchical schematic outlines the core organizational architecture adopted throughout this review to unify scattered research on ncRNA-mediated host–virus crosstalk.

## 2 NcRNA-mediated host antiviral responses against viral replication

### 2.1 Upregulated antiviral ncRNAs post-viral infection

A panel of host miRNAs is markedly upregulated upon IAV infection, and these molecules exert robust antiviral activity through diverse molecular mechanisms: targeting proviral host factors to restore innate immune signaling pathways, directly interacting with viral genomic regions to mediate viral RNA degradation and suppress viral protein synthesis, and inhibiting viral replication-associated host or viral proteins (e.g., viral RNA-dependent RNA polymerase, RdRp) to impair viral replication kinetics. miR-200c targets the proviral host factor CNTN1 to reduce its expression, which in turn blocks CNTN1-induced MAVS K63-linked deubiquitination and degradation, restoring the RIG-I/MAVS signaling pathway to exert antiviral activity (34). miR-323 and miR-654 bind to the conserved region of the IAV PB1 gene and mediate the degradation of its mRNA (rather than translation repression) to downregulate PB1 expression and inhibit viral replication (35). miR-let-7c forms a perfect complementary combination with the 3’ UTR of IAV M1 (+) cRNA, triggering its degradation to reduce M1 expression at both the nucleic acid and protein levels and constrain viral replication (36). Additionally, miR-101 is induced at the late stage of IAV infection to target and downregulate mTOR transcripts, thereby suppressing viral NP protein synthesis and reducing viral titers (37). miR-203 is upregulated by IAV via type I IFN-mediated transcriptional activation and DNMT1-dependent promoter demethylation, and exerts antiviral effects by targeting DR1 to abrogate its pro-viral suppression of IFN response and enhancement of viral RdRp activity (38).

Increasing evidence has revealed that host lncRNAs act as essential regulators of antiviral immunity during IAV infection. A subset of lncRNAs primarily targets the viral replication cycle. LncRNA-45 and lncRNA-61 translocate from the nucleus to the cytoplasm after infection and inhibit viral polymerase activity as well as nuclear accumulation of viral NP and PA proteins through their stem-ring/long-ring structural domains, thereby broadly suppressing IAV replication (39, 40). LncRNA-GBP1P1 is induced by the JAK/STAT signaling pathway and functions as a molecular sponge to bind and sequester DHX9, reducing its utilization by viral mRNAs (41). LncRNA-LINC01197 is induced by IAV infection, and its expression is regulated by the NF-κB pathway. It directly interacts with cytoplasmic PABPC1 to act as a protein decoy, competitively binding PABPC1 and abrogating its association with IAV mRNAs and NS1 protein, thereby inhibiting the translation of viral mRNAs and exerting anti-IAV effects at the translational stage of the viral replication cycle (42). In contrast, most infection-induced lncRNAs exert their effects by regulating innate immune signaling pathways. LncRNA-01615 is significantly upregulated upon IAV infection, and it positively regulates the expression of IFN-β, IL-28A, IL-29, and downstream ISGs, thereby potentiating the host innate antiviral response and suppressing IAV replication (43). LncRNA-155 is encoded by MIR155HG, and its expression is regulated by the RIG-I/TLR3-dependent NF-κB/IFN-β pathway. This lncRNA inhibits the expression of the negative regulator PTP1B, thereby relieving PTP1B-mediated suppression of type I IFN signaling and consequently enhancing the production of IFN-β and downstream ISGs to potentiate the host antiviral response (44). LncRNA-9101 is induced by IAV infection in chicken-derived cells. It positively regulates the TLR7/MDA5 signaling pathway and promotes the phosphorylation and activation of IRF7, thereby enhancing the transcriptional expression of IFN-β and downstream ISGs and effectively suppressing IAV replication (45). LncRNA-AVAN has dual nuclear and cytoplasmic functions, promoting FOXO3a transcription in the nucleus to enhance neutrophil chemotaxis, and binding TRIM25 in the cytoplasm to enhance K63-linked ubiquitination of RIG-I, amplifying type I IFN and ISGs expression (46). LncRNA-IFITM4P acts as a ceRNA to sponge miR-24-3p and stabilize IFITM1/2/3 transcripts (47). LncRNA-ISG20 sponges miR-326 through a ceRNA mechanism to relieve translational repression of ISG20 and strengthen antiviral signals (48). LncRNA-IVRPIE is upregulated upon IAV infection. It specifically binds to hnRNP U in the nucleus, thereby regulating histone modifications at the transcription start sites of IFN-β and multiple ISGs (including IFIT1 and ISG15) by increasing H3K4me3 and decreasing H3K27me3, which in turn upregulates the transcription of these genes and activates host antiviral immune signals (49). LncRNA-LINC02574 is induced by IAV infection in a RIG-I-dependent manner, with its expression regulated by IFNAR1. It maintains the expression of RIG-I, TLR3, and MDA5 and promotes IRF3 phosphorylation and STAT1 activation, thereby positively regulating type I/III IFN responses (50). The expression of lncRNA-NEAT1 is regulated by STAT3, MDA5, and TLR3/NF-κB pathways. It enhances IFN-mediated antiviral response by promoting TBK1 activation to suppress IAV replication (51). LncRNA-RDUR is induced by the RIG-I/NF-κB pathway. It binds to ILF2/ILF3 to promote IRF3 phosphorylation, thereby upregulating IFN-β expression, activating STAT1 signaling, and inducing ISGs production to suppress IAV replication, while negatively feedback inhibiting NF-κB to balance inflammation and assist antiviral responses (52). As an IFN-inducible lncRNA, lncRNA-RPS6P3 binds to NP to inhibit its oligomerization and vRNP activity and simultaneously binds to viral NS1 protein to abrogate its suppression on RIG-I, restoring RIG-I-mediated IFN-β production and activating immune pathways (53). LncRNA-SAAL is upregulated upon IAV infection. It promotes the transcriptional activation of IFN-β and downstream ISGs by upregulating Serpina3i expression, thereby potentiating the host antiviral response to suppress IAV replication (54). LncRNA-PINK1-2:5 is a nucleus-localized, type I IFN-independent lncRNA that exerts broad-spectrum anti-IAV effects by upregulating the host restriction factor TXNIP. Whether its biological functions are associated with antiviral innate immune signaling pathways remains to be further investigated (55). Collectively, these IAV infection-upregulated host miRNAs and lncRNAs exert potent, multifaceted antiviral effects via distinct complementary strategies: targeting proviral host factors to relieve immune signaling repression, directly targeting viral components to disrupt replication, modulating host innate immune signaling pathways to amplify antiviral responses and balance inflammation, and sequestering viral/host functional proteins or acting as ceRNAs to post-transcriptionally regulate antiviral factor expression, thus forming an elaborate host ncRNA regulatory network for effective IAV defense.

CircRNAs are covalently closed, non-polyadenylated transcripts lacking 5′ caps and 3′ poly(A) tails, which exert multifaceted regulatory roles during viral infection, most prominently via miRNA sequestration. Beyond miRNAs and lncRNAs, host circRNAs have also emerged as crucial regulators of IAV infection, with multiple upregulated circRNAs exerting antiviral effects through distinct molecular mechanisms. CircRNA-0008085 is induced by IAV infection in a JAK/STAT pathway-dependent manner, and it acts as an endogenous sponge for miR-146a-5p to suppress its activity, thereby upregulating the expression of TRAF6 and further inhibiting IAV replication (56). CircRNA-0082633 is another IAV-induced circRNA whose expression is regulated by JAK/STAT pathway activation. Its overexpression restrains IAV proliferation by enhancing ISRE promoter activity and IFN-β mRNA levels, thus activating type I IFN signaling (57). CircRNA-AIVR, an intronic circRNA upregulated in IAV-infected A549 cells, localizes predominantly in the cytoplasm and functions as a miRNA sponge to sequester miRNAs targeting CREBBP mRNA. This interaction elevates CREBBP expression, which in turn promotes IFN-β production and exerts antiviral effects, while silencing of circRNA-AIVR significantly facilitates IAV replication (58). CircRNA-CBP is markedly upregulated upon IAV infection or IFN treatment, and it exerts multi-faceted antiviral effects. It binds to NP to reduce its interaction with PB1 and PB2, thereby blocking vRNP activity. It also interacts with NS1 to alleviate NS1-mediated inhibition of antiviral immunity, and binds to G3BP1 to enhance its oligomerization, promoting stress granule formation and IFN-β transcription (59). CircRNA-MYO9A, a novel differentially expressed circRNA during IAV infection, acts as a ceRNA by sponging miR-6059-3p to upregulate SERPINE1/PAI-1 expression. This upregulation restricts IAV haemagglutinin cleavage, reduces the infectivity of progeny viruses, and inhibits viral replication in the lungs of infected mice, potentially improving their survival rate (60). CircRNA-VAMP3 is substantially upregulated after IAV infection or IFN stimulation, with its biogenesis promoted by QKI-5 (induced by IAV and IFN-β). VAMP3 not only inhibits IAV replication *in vitro* and restricts viral replication and pathogenesis in mice but also exerts dual antiviral mechanisms. It binds to NP to reduce its interaction with PB1, PB2, or vRNA, impairing vRNP complex activity, and interacts with NS1 to alleviate its inhibitory effect on RIG-I or TRIM25, restoring IFN-β activation to reinforce innate antiviral immunity (61). In brief, these findings highlight the diverse and specific antiviral strategies employed by circRNAs in modulating IAV replication and host immune responses.

In addition to circRNAs associated with IAV infection, host ncRNAs also display characteristic expression changes during the infection of DENV, HCV, and ZIKV and participate in antiviral immune regulation. During DENV infection, the expression of miR-30e* is induced by the virus. miR-30e* activates NF-κB signaling by targeting IκBα and disrupting the negative feedback loop of the NF-κB pathway, which further promotes the expression of IFN-β and downstream ISGs to suppress viral replication (62). miR-548g-3p binds directly to the stem-loop A region within the 5’UTR of the DENV genome, interferes with viral translation, and inhibits the proliferation of multiple DENV serotypes (63). HCV infection also induces multiple host miRNAs to exert antiviral functions. miR-125b-5p is highly expressed in HCV-infected cells and the serum of patients with HCV infection. It directly targets the 3’UTR of HuR to downregulate its protein expression at the translational level. This regulatory axis potently suppresses HCV replication, while knockdown of miR-125b-5p restores HuR expression and reverses the inhibitory effect on viral replication (64). miR-221 is significantly upregulated in infection models and enhances the antiviral effect of IFN by targeting SOCS1 and SOCS3, which are negative regulators of the IFN-JAK-STAT pathway (65). miR-27a is specifically upregulated in HCV-infected liver via C/EBPα, directly targets RXRα and ABCA1, modulates lipid metabolism to reduce HCV particle infectivity, enhances IFN signaling, and forms a negative feedback loop to inhibit HCV replication (66). At the level of lncRNAs, the expression of lncRNA-BST2–2 is regulated by the IFN pathway. It enhances interferon production by binding to IRF3 and promoting its phosphorylation and nuclear translocation, thereby inhibiting the replication of multiple viruses, including HCV (15). LncRNA-GAS5 expression increases during infection. It binds to the HCV NS3 protein through its 5’-terminal sequence and inhibits protein function, thus negatively regulating viral replication (25). LncRNA-ITPRIP-1 is induced by viral infection and binds to the C-terminus of MDA5 to promote its oligomerization and activation, thereby enhancing the virus-triggered IFN signaling pathway and boosting the host innate immune responses to viral infection for antiviral effects (67). Furthermore, lncRNA-OASL-IT1 is induced and upregulated upon ZIKV infection in an IPS-1- and IFNAR1-dependent manner, elevates cellular IFN levels and the activation of innate immune signaling pathways, and forms a positive feedback regulatory loop with IFNs to facilitate the establishment of the host antiviral defense system (68). These ncRNAs collectively form an important regulatory network for host defense against flavivirus infection.

In retroviral infections, host-encoded ncRNAs also exert pivotal regulatory functions in the innate defense. HIV-1 infection markedly upregulates the expression of host miR-186, miR-210, and miR-222, which collectively downregulate the expression of host target genes Dicer1, HRB, and HIV-EP2, and effectively inhibit HIV-1 replication by suppressing viral gene expression, viral RNA nucleocytoplasmic transport, and miRNA biogenesis (69). miR-198 is an antiviral microRNA upregulated upon HIV-1 infection, which directly targets the major functional site 6502 in the 3’UTR of Cyclin T1 mRNA and represses its protein expression at the translational level. As an essential cofactor for HIV-1 Tat-mediated transactivation of viral LTR transcription, Cyclin T1 depletion by miR-198 constitutes a direct antiviral pathway that inhibits HIV-1 replication (70). Together, these upregulated miRNAs form a multifaceted host antiviral regulatory network that restricts HIV-1 replication through distinct molecular pathways, highlighting the critical role of host ncRNAs in the innate defense against retroviral infection.

### 2.2 Downregulated proviral ncRNAs post-viral infection

In addition to upregulating antiviral ncRNAs, the host can initiate antiviral defenses by downregulating endogenous proviral ncRNAs, enhancing host antiviral capacity by weakening the proviral effects of these RNAs and relieving their inhibition on antiviral immunity. During IAV infection, the significant downregulation of multiple proviral miRNAs is a key component of the host antiviral responses. The downregulation of miR-194 relieves its targeted inhibition of FGF2, restoring FGF2-mediated antiviral signaling, while the miRNA itself promotes viral replication by inhibiting FGF2 and type I IFN production (71). miR-21-3p is downregulated in infected cells, weakening its proviral effect of targeting HDAC8 and suppressing FGF2 and the type I IFN response (72, 73). miR-26a-5p is downregulated upon H5N1 infection, relieving its inhibition on the MDA5 pathway and related molecules such as MAVS, IRF7, and NF-κB to enhance host antiviral capacity (74). The downregulation of miR-302c releases its inhibition on NIK, restoring NF-κB nuclear translocation and IFN-β expression to restrict viral replication (75). miR-340-5p is downregulated following IAV and other RNA virus infections, reducing its inhibitory effect on RIG-I and OAS2 and enhancing the host’s antiviral defense function (76). miR-4276 is rapidly downregulated in the early stage of infection, limiting viral propagation by increasing COX6C expression and activating caspase-9-mediated apoptosis (77). The downregulation of miR-93 relieves its inhibition on JAK1, strengthening IFN effector signaling to suppress viral infection (78). Similarly, several proviral lncRNAs are also downregulated upon IAV infection. The downregulation of lncRNA-AROD abrogates its ceRNA function to sequester miR-324-5p, leading to CUEDC2 downregulation, which relieves the CUEDC2-mediated inhibition of IFN-β and ISGs and thus reduces viral replication and pathogenicity (79). The downregulation of lncRNA-NRAV reduces its epigenetic inhibition on the transcription of ISGs such as IFITM3 and MxA, helping the host initiate antiviral innate immunity (16). The downregulation of lncRNA-THRIL relieves its inhibition on IRF3 activation and type I and III IFN responses, enhancing host defense to suppress viral replication (80).

Host downregulation of proviral ncRNAs represents a conserved antiviral strategy across diverse viral infections. For instance, during DENV infection, miR-378 is downregulated in patients, relieving its inhibition on GrzB in NK cells and enhancing the inhibitory effect of GrzB on viral replication (81). In HCV and other IFN-susceptible viral infections, miR-1225-3p is downregulated through the type I IFN-JAK-STAT pathway, reducing its targeted inhibition on GAB3 and enhancing IFN-mediated antiviral capacity (82). With respect to HIV-1 infection, the downregulation of miR-34c-5p weakens its proviral role. This miRNA targets and inhibits KAT2B/PCAF (a transcriptional co-activator required for HIV-1 Tat-dependent LTR transcription) and regulates naive CD4+ T-cell activation and TCR signaling, thus its downregulation is an important part of the host antiviral responses (83). In ZIKV infection, circRNA-0007321 is significantly downregulated, restricting viral replication by regulating the miR-492/NFKBID/NF-κB pathway (84). Taken together, these findings demonstrate that downregulation of proviral ncRNAs is a broad and critical host strategy to counteract viral replication by neutralizing the immune-inhibitory and replication-promoting functions of these RNAs.

Collectively, this section elaborates two evolutionarily conserved and mutually complementary antiviral defense axes shaped by dynamic remodeling of host ncRNAs upon viral infection. On one front, infection triggers robust induction of diverse miRNAs, lncRNAs and circRNAs, which confer broad antiviral activity via multiple independent mechanisms. On the other hand, hosts deploy a relatively underappreciated protective strategy by repressing endogenous proviral ncRNAs. Dampening the levels of these immune-inhibitory RNAs relieves constitutive suppression of RIG-I, IRF and ISG signaling, thereby strengthening cellular intrinsic antiviral competence. Shared core regulatory logic can be observed across IAV, flaviviruses including DENV, HCV and ZIKV, as well as HIV-1 infections. Host ncRNAs converge on the conserved interferon signaling cascade to calibrate host immunity, whereas clear virus-specific distinctions remain in their molecular targets and functional magnitude. Aligned with the four-category classification system shown in **Figure 1** and visually illustrated in **Figure 2**, bidirectional modulation of ncRNA abundance constitutes the core host ncRNA-mediated antiviral program. A central takeaway derived from the compiled body of evidence is that hosts rely on both upregulation of antiviral ncRNAs and downregulation of proviral ncRNAs to mount comprehensive antiviral responses, rather than relying merely on the induction of antiviral ncRNAs.

**Figure 2:**
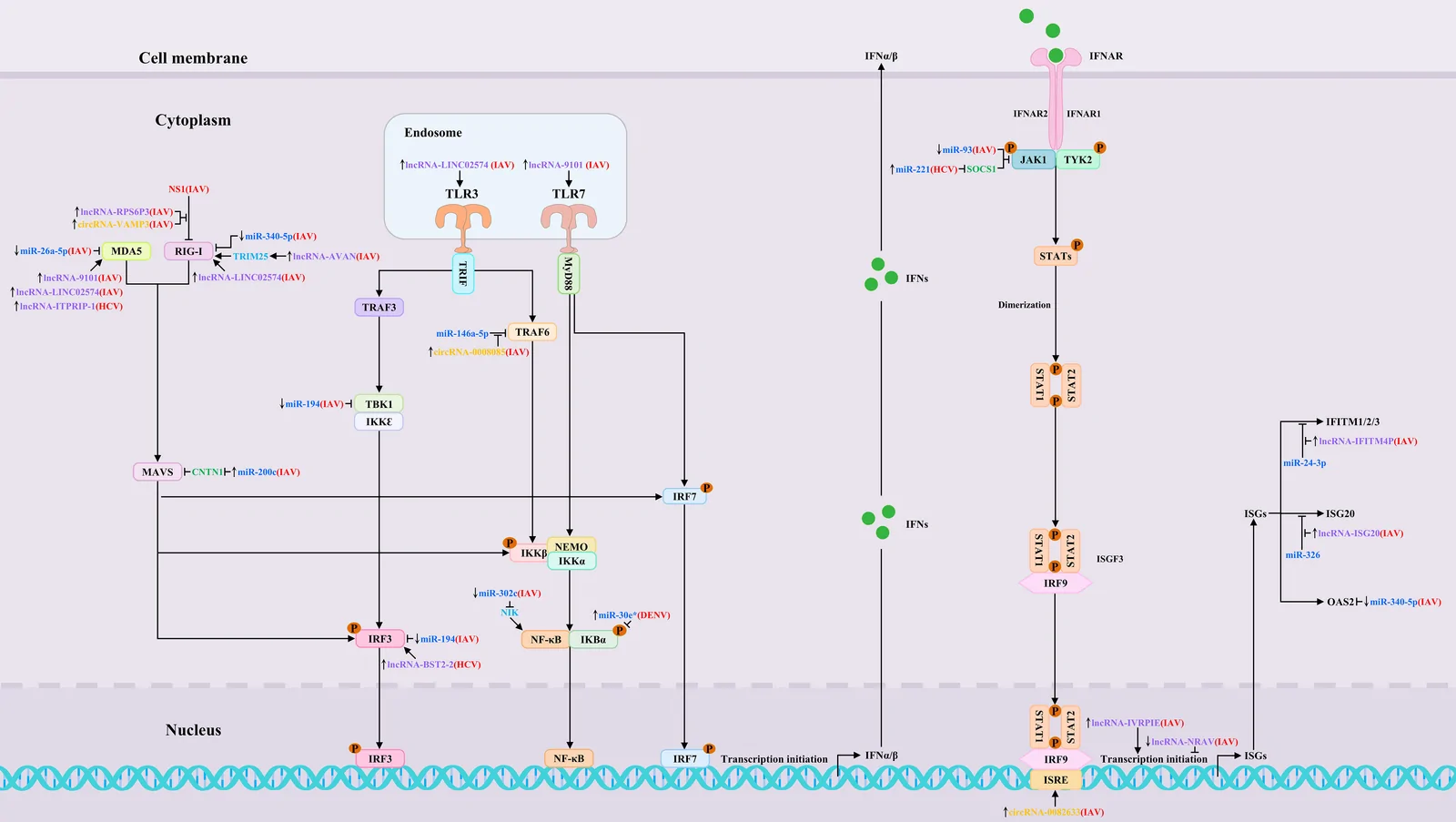
Host ncRNA-mediated antiviral innate immune signaling pathways against viral infection. Upon viral infection, hosts execute two-layered antiviral defense strategies by dynamically remodeling ncRNA expression: (1) upregulating antiviral ncRNAs to activate innate immune signaling and restrict viral proliferation; (2) downregulating proviral ncRNAs to relieve immune inhibition and restore antiviral competence. These ncRNAs target PRRs, interferon signaling, ISGs, host restriction factors, proviral host factors, and other key molecules to collectively establish a robust antiviral state.

## 3 Viruses hijack ncRNAs to facilitate viral replication

As the most well-studied virus in terms of ncRNA hijacking, IAV first exploits host miRNAs to lay the foundation for efficient replication, given that miRNAs act as the earliest and most extensively regulated ncRNA class during IAV infection. Specifically, the core mechanisms of IAV-associated miRNAs are as follows: miR-125a/b is significantly upregulated upon IAV infection in airway epithelial cells and experimental models, directly targeting the 3′-UTR of MAVS to suppress its expression. This inhibition impairs RIG-I/IRF3-mediated induction of type I/III IFNs and downstream ISGs, thereby weakening host antiviral defense and promoting IAV replication (23). IAV infection induces miR-1290 expression through ERK pathway activation. This miRNA directly targets and inhibits the host VIM gene, and downregulation of VIM increases nuclear retention of viral vRNP and polymerase activity, thereby enhancing viral replication (85). IAV infection upregulates miR-132-3p in a dose- and time-dependent manner, which directly targets IRF1 to weaken type I IFN and downstream ISGs expression, suppressing innate immunity and promoting IAV replication (86). miR-146a is significantly upregulated by IAV infection, directly targeting TRAF6 to reduce IFN-β and ISGs expression, impairing antiviral immunity and promoting IAV replication (19). IAV (H1N1/PR8) infection upregulates IFN-induced miR-193b-5p, which targets occludin to reduce its protein level, disrupting alveolar-capillary barrier integrity and promoting viral replication (87). IAV infection activates the ERK/p38 MAPK pathway, inducing the transcription factor CREB to bind the miR-200b-3p promoter and upregulate its expression. miR-200b-3p directly targets the 3’ UTR of TBK1 to inhibit its expression, impairing IRF3- and NF-κB-mediated type I IFN production and thereby promoting IAV replication (88). miR-34c is upregulated by IAV infection, promoting the transcription and expression of host kinase PLK4 (essential for IAV replication) to enhance viral propagation (89). miR-4776 is upregulated within 1 hour of IAV infection, directly targeting the 3’ UTR of NFKBIB to relieve its retention of NF-κB, indirectly regulating NF-κB activity to promote IAV replication (90). miR-664a-3p is upregulated by IAV (predominantly H7N9) infection, directly targeting the key antiviral factor LIF to suppress its expression and enhance viral replication, while its inhibition rescues LIF function and confers broad-spectrum antiviral activity against H7N9, H1N1, and H3N2 (91). IAV infection early induces miR-9 expression in A549 cells, which targets MCPIP1 (a virus RNA-degrading ribonuclease) to abrogate its antiviral effect, promoting IAV gene expression and progeny virus production (92). Collectively, IAV hijacks diverse miRNAs through precise regulatory mechanisms to modulate host antiviral immunity and cellular functions, creating a favorable initial environment for viral replication, which is complemented by the regulatory role of lncRNAs.

Following the regulatory effects of miRNAs, lncRNAs act as important host regulatory molecules induced by IAV infection and key mediators of viral replication, which further establish a cellular microenvironment permissive for IAV replication via specific regulatory mechanisms. LncRNA-8475 is significantly upregulated following H9N2 IAV infection. It inhibits the TLR3- and TLR7-mediated innate immune signaling pathway, thereby blocking STAT1 activation and directly targeting and suppressing the expression of key type I/III IFNs and downstream ISGs, ultimately weakening host antiviral defense and promoting IAV replication (45). LncRNA-ACOD1 is inducible by IAV and other viruses (not regulated by type I IFN), localized in the cytoplasm, and directly binds the substrate-binding region of metabolic enzyme GOT2 to enhance its catalytic activity, regulating host metabolic networks via an IFN-I-IRF3-independent pathway to provide material and energy support for viral replication (93). LncRNA-ALOX12 is specifically upregulated by IAV infection, binding to IAV RNA polymerase subunit PB2 to maintain its interaction with importin-α/β, ensuring PB2 nuclear import and efficient viral RNA synthesis to promote IAV replication. Avian influenza viruses require PB2 mutations to hijack human ALOX12, a species-specific regulation that acts as a barrier for cross-species transmission (27). Notably, certain lncRNAs harbor open reading frames and produce small functional micropeptides that participate in viral regulatory networks. LncRNA-GAS5 is significantly upregulated by IAV infection, type I/III IFNs, and IL-6 stimulation, exerting pro-viral effects via its encoded 50-amino acid micropeptide GAS5-P50 that interacts with Wnt signaling negative regulator NOTUM to activate the Wnt/β-catenin pathway and promote IAV replication (26). LncRNA-IPAN is specifically induced by IAV infection (IFN-independent), binding to IAV RNA polymerase subunit PB1 to both stabilize PB1 and block its degradation by the RIG-I-TRIM25 complex (RIG-I mediates degradation via “effector-like” activity independent of its classical signaling pathway), helping IAV evade immune surveillance and promote replication (94, 95). LncRNA-MALAT1 is significantly upregulated by IAV infection *in vitro* and *in vivo* (IFN-independent, regulated by the NF-κB/IL-6/STAT3 pathway), while also inducing the endogenous upregulation of its encoded 52-amino acid micropeptide miPEP-52. MALAT1 exerts core proviral effects via miPEP-52, with both inhibiting host innate immune responses against IAV to promote replication (96). LncRNA-MxA, an ISG, is significantly upregulated by IAV infection. Its overexpression promotes IAV replication while knockdown inhibits it, forming an RNA–DNA triplex with the IFN-β promoter to interfere with IRF3 and p65 binding, thereby suppressing RIG-I-mediated IFN-β transcriptional activation and negatively regulating the RLR pathway antiviral response, participating in a negative feedback loop to maintain immune homeostasis (97). LncRNA-NSPL is highly expressed in monocytes of IAV-infected patients, directly binding RIG-I during the late stage of IAV infection to block its interaction with E3 ligase TRIM25, reducing TRIM25-mediated K63-linked ubiquitination of RIG-I and limiting the production of downstream antiviral mediators to facilitate immune escape and viral replication (98). LncRNA-PAAN (human-derived) is specifically upregulated by IAV infection, binding to the key component PA protein of the IAV RdRp complex to facilitate the assembly of functional RdRp and ensure efficient viral RNA synthesis, thereby promoting IAV replication (99). LncRNA-PCBP1-AS1 and its encoded 110-amino acid micropeptide PESP are both significantly upregulated by IAV infection and type I/III IFNs, with both promoting IAV replication. PESP is the key functional molecule that enhances IAV-induced autophagy by upregulating ATG7 and maintains stability by binding HSP90AA1 to facilitate viral proliferation (100). LncRNA-TSPOAP1-AS1 is significantly upregulated by IAV infection in a dose- and time-dependent manner, with its expression regulated by NF-κB signaling and its nucleocytoplasmic ratio increased post-infection. It negatively modulates IAV-induced IFN-β transcription, ISRE activation, and downstream ISGs expression to weaken antiviral immunity and promote IAV replication (101). LncRNA-up4 is significantly upregulated by H9N2 IAV infection via the pattern recognition receptor (PRR)-dependent NF-κB signaling pathway, predominantly localized in the nucleus, and suppresses IFN-β, Mx1, and OAS1 expression to promote IAV replication (28). LncRNA-USP30-AS1 is induced by IAV via the JAK-STAT signaling pathway, directly binding to PHB1 to on the one hand to sequester PHB1 away from the E3 ubiquitin ligase TRIM21 to protect its protein stability, and on the other hand serve as a molecular scaffold to enhance the interaction between PHB1 and IRF3, thereby impeding the nuclear import of IRF3 and thus inhibiting antiviral signaling to promote IAV replication (102). Together with miRNAs, lncRNAs form a synergistic regulatory network that comprehensively modulates host cellular processes, which is further supplemented by the regulatory effects of circRNAs and vtRNAs.

In addition to miRNAs and lncRNAs, IAV also hijacks circRNAs and vtRNAs to further facilitate its replication, forming a comprehensive regulatory network of host ncRNAs that covers all major ncRNA classes. Transitioning to circRNAs, these covalently closed ncRNAs serve as key IAV-induced regulatory modules that contribute to viral replication through distinct mechanisms. CircRNA-0050463, predominantly localized in the cytoplasm, is upregulated by IAV infection via NF-κB signaling and functions as an endogenous miR-33b-5p sponge, sequestering and inhibiting miR-33b-5p activity to increase the expression of EEF1A1, which in turn facilitates IAV replication (103). CircRNA-GATAD2A is specifically induced upon H1N1 infection in a dose-dependent manner, and it promotes viral replication by inhibiting VPS34-dependent autophagy. Mechanistically, GATAD2A interacts with VPS34 (a key regulator of autophagy) to suppress autophagic activity, as evidenced by reduced LC3-II levels and accumulated P62. Notably, its pro-viral effect is abrogated in VPS34-knockout cells, confirming that GATAD2A exerts its function through the VPS34-mediated autophagic pathway (104). CircRNA-MerTK, derived from the MerTK pre-mRNA, is highly induced by IAV infection *in vivo*, with its expression regulated by RIG-I/IFN signaling. It suppresses the production of type I IFNs and ISGs, thereby weakening host antiviral immunity and promoting IAV replication (105). Notably, vtRNAs represent a unique class of polymerase III-transcribed ncRNAs with distinct immune-regulatory functions that have only been characterized in IAV infection to date. Finally, vtRNAs are robustly induced by the IAV NS1 protein in both A549 cells and mouse lungs, and their upregulation is essential for efficient viral replication. Specifically, vtRNAs promote viral propagation by repressing the activation of PKR and the subsequent antiviral IFN response, and they are also required for the NS1-mediated suppression of PKR, representing a conserved mechanism exploited by multiple viruses to circumvent PKR-mediated innate immunity (106). In conclusion, these findings reveal that IAV subverts nearly all major classes of host ncRNAs through diverse, specific mechanisms, targeting and regulating proviral host factors and host restriction factors, rewiring host metabolic networks and cellular processes (e.g., autophagy), suppressing host innate immune responses, sustaining viral gene expression and replication machinery assembly, and even encoding functional micropeptides to facilitate viral propagation, creating a replication-permissive intracellular milieu that supports efficient viral infection and propagation, laying a foundation for understanding the ncRNA-mediated regulatory mechanisms of other viruses.

Extending beyond IAV, flaviviruses, a major group of human-pathogenic RNA viruses within the Flaviviridae family, also hijack diverse host ncRNAs through sophisticated regulatory mechanisms to subvert host antiviral defenses and create a permissive microenvironment for their replication, with distinct regulatory principles among different flavivirus species. For DENV, miR-146a is significantly upregulated in human primary monocytes and THP-1 cells upon infection, which directly targets and inhibits TRAF6 to impair IFN-β production and its downstream antiviral effects, thereby promoting DENV2 replication (20). LncRNA-SUN2-AS1 is a conserved proviral lncRNA shared by flaviviruses, including DENV and ZIKV, whose expression is significantly upregulated upon flavivirus infection. This lncRNA directly represses the transcription of ISGs, thereby impairing the host antiviral immunity and facilitating the infection and replication of flaviviruses such as DENV and ZIKV. Importantly, its proviral effect is flavivirus-specific, with no discernible impact on non-flaviviral infections, including vesicular stomatitis virus (VSV) and herpes simplex virus type 1 (HSV-1) (107). With respect to HCV, miR-125a is significantly upregulated in hepatocytes and patient serum upon infection, which directly targets and inhibits MAVS and TRAF6 to weaken the type I IFN signaling pathway, helping HCV escape host innate immune surveillance for immune escape and persistent infection (24). miR-130a is upregulated in hepatocytes after HCV infection, which directly targets and inhibits IFITM1 to reduce its expression, impairing IFITM1-mediated antiviral effects and promoting HCV replication (108). miR-135a is significantly upregulated in *in vitro* cultured hepatocytes and liver tissues of patients with chronic hepatitis C, which specifically targets the HCV genome replication stage without affecting other life cycle links such as viral entry and translation. It specifically binds to the 3’UTR of MyD88 through its seed sequence to directly inhibit MyD88 expression at the transcriptional and translational levels, thereby blocking the initiation and transmission of antiviral innate immune signals and weakening the host innate antiviral responses against HCV (109). miR-208b and miR-499a-5p are abnormally highly expressed in hepatocytes induced by HCV infection, both directly targeting and inhibiting the expression of the type I IFN receptor subunit IFNAR1 to weaken the transmission of the type I IFN signaling pathway in hepatocytes and reduce its mediated antiviral response. They also inhibit the type III IFN family members IFNL2/IFNL3, achieving dual inhibition of type I and III IFN pathways to help HCV establish persistent infection (110). HCV activates the PKCϵ/JNK/c-Jun and PKCα/ERK/c-Fos signaling cascades through viral proteins to regulate the transcription factor AP-1-mediated induction of miR-21, which is significantly upregulated by HCV infection and directly targets and inhibits the core molecules MyD88 and IRAK1 of the PRR pathway to block HCV-triggered type I IFN production and weaken the host innate antiviral immune response (111). miR-21-5p is significantly upregulated in HCV-infected hepatocytes and patient liver tissues, with its expression and activity specifically induced by the HCV-3a core protein. This miRNA directly targets and inhibits the expression of the tumor suppressor PTEN, thereby relieving the restriction of PTEN on the HCV life cycle and significantly promoting HCV genomic replication and the release of infectious viral particles (112). miR-215, associated with HCV infection, significantly promotes viral replication by directly targeting and inhibiting TRIM22. Inhibition of TRIM22 by miR-215 blocks the activation of the NF-κB signaling pathway, thereby weakening the antiviral response mediated by this pathway, and restoration of TRIM22 expression can reverse the pro-replication effect of miR-215 on HCV (113). miR-373 is significantly upregulated in HCV-infected hepatocytes and patient liver tissues, impairing the host antiviral response through multi-target inhibition of the type I IFN signaling pathway. It directly targets and inhibits JAK1 and IRF9, thereby suppressing STAT1 phosphorylation and hindering the formation of the ISGF3 complex (114). Concurrently, it targets and inhibits IRF5, reducing the expression of ISGs such as PKR, OAS, and MxA. These dual mechanisms collectively weaken the host antiviral defense, creating a favorable environment for HCV replication (115). miR-4423-3p is significantly upregulated in HCV-infected hepatocytes, directly binding to RIG-I mRNA to inhibit its expression and block the activation of the RIG-I/IFN signaling pathway, thereby markedly promoting the infection process of HCV in hepatocytes (116). miR-758 is significantly upregulated in patients and hepatocytes upon HCV infection, with the HCV core protein being the key mediator. It directly targets and inhibits the expression of PRRs TLR3 and TLR7, blocking the activation of TLR3/TLR7 signaling triggered by viral RNA and reducing the production of type I IFN-α and IFN-β (117). miR-93-5p is significantly upregulated in patient serum and hepatocytes by the HCV-1b core protein. It directly targets and inhibits the type I IFN receptor subunit IFNAR1, blocking IFNAR1-mediated STAT1 phosphorylation and inactivating the IFN signaling pathway (118). LncRNA-ATV is upregulated upon type I/III IFN stimulation and viral infection and is localized in the cytoplasm. It binds to the key innate immune molecule RIG-I to inhibit the activation of the RIG-I antiviral signaling pathway and the IFN effect pathway, ultimately facilitating HCV replication (119). LncRNA-CMPK2 is significantly upregulated in the liver tissues of HCV-infected patients. As an ISG dependent on the JAK-STAT signaling pathway, it transcriptionally represses the expression of antiviral ISGs, including Viperin, thereby impairing host antiviral defenses and facilitating HCV replication (17). LncRNA-EGOT is an HCV-inducible lncRNA whose expression is regulated by the RIG-I/PKR-NF-κB pathway activated by HCV RNA. As a negative regulator of the antiviral pathway, its knockdown markedly upregulates ISGs, including ISG15, and potently inhibits HCV replication (120). LncRNA-HULC is significantly upregulated by HCV infection *in vitro* and *in vivo*, with the HCV non-structural protein NS5A directly promoting HULC transcription. HULC positively regulates HCV replication by regulating the viral internal ribosome entry site-mediated translation process, and HCV eradication significantly reduces HULC levels in patient liver tissue and blood (121). LncRNA-ITM2C-1 is markedly upregulated in hepatocytes during the early stage of HCV infection. It promotes the mRNA expression of the adjacent gene GPR55, which in turn downregulates the expression of ISGs, including ISG15, Mx1, and IFITM1, thus facilitating HCV replication (122). LncRNA-UCA1 is upregulated in Huh7.5 cells in a time- and dose-dependent manner upon HCV infection and is also highly expressed in HCV-infected individuals. As a ceRNA, it sponges miR-145-5p to relieve its targeted silencing of SOCS7 mRNA, thereby upregulating SOCS7 protein expression, which inhibits the host IFN response and promotes HCV replication (123). For ZIKV, miR-103a-3p is upregulated in A549 cells upon infection, which significantly promotes ZIKV NS5 mRNA transcription and NS1 protein expression. It directly targets and inhibits OTUD4 expression to activate the p38 MAPK signaling pathway, and activation of the p38 MAPK pathway is critical for the pro-ZIKV replication effect of miR-103a-3p (124). The NS1 protein of ZIKV upregulates miR-146a in human microglial cells, which targets and inhibits the expression of TRAF6 as well as STAT1 phosphorylation, and downregulates IFN-β expression, thereby blocking the type I IFN antiviral signaling pathway and impairing host antiviral defenses to facilitate ZIKV replication (21). ZIKV infection upregulates the expression of host lncRNA-LINC08148, which binds to the transcription factor SP1 and promotes the transcription of its target gene Src. As a key initiator of caveola-mediated endocytosis, Src enhances the caveola-dependent endocytic entry of ZIKV, thereby facilitating ZIKV replication in host cells (125). To sum up, flaviviruses hijack a diverse array of host ncRNAs via regulatory mechanisms with both species-specific and conserved features, targeting host innate immune signaling pathways, viral life cycle processes, and host functional molecules, and employing the ceRNA-sponge mechanism to comprehensively disrupt host antiviral defenses and modulate cellular physiological processes, ultimately facilitating viral replication and persistent infection.

Beyond flaviviruses, HIV-1 has also evolved sophisticated strategies to hijack host miRNAs and lncRNAs, using cell-type-specific regulatory principles to subvert host antiviral defenses, modulate viral transcription and latency, and ultimately promote its replication and persistence in target cells. HIV-1 infection upregulates miR-146a in infected cells, which directly targets TRAF6 and IRAK1 to suppress host innate immune responses and negatively regulate the expression of NF-κB-related cytokines and ISGs, thereby facilitating viral replication (22). miR-197-3p is significantly elevated in multiple cell types upon HIV-1 infection. It directly targets and inhibits DDX52 to relieve the suppression of the HIV-1 infectivity factor Vif, thus markedly enhancing the infectivity of progeny virions (126). The HIV-1 accessory protein Vpu hijacks the host β-catenin pathway to induce the expression of miR-25 and miR-93 in macrophages. Both miRNAs target and degrade MARCH1 mRNA, relieving its restriction on viral envelope glycoprotein incorporation and supporting the production of infectious HIV-1 particles (127). HIV-1 upregulates miR-422a in primary CD4+ T cells via the accessory protein Nef. This miRNA directly targets MECP2 to promote viral replication and also regulates the IFN-α-mediated antiviral pathway, with its expression level correlated with viral load in infected individuals (128). HIV-1 infection increases the expression of lncRNA-HEAL in multiple cell types. HEAL forms a complex with the RNA-binding protein FUS and broadly enhances the replication of diverse HIV-1 strains by promoting HIV promoter activity and CDK2 expression (129). LncRNA-MALAT1 is markedly upregulated in HIV-1-infected macrophages and CD4+ T cells and promotes viral replication through distinct mechanisms. In macrophages, it sponges miR-145-5p to relieve its inhibition on CHCHD2, thereby downregulating IRF7 and STAT2 to suppress innate immunity (130). It also sponges miR-150-5p to relieve its inhibition on SOCS1, leading to SOCS1 upregulation that in turn facilitates viral replication (131). In CD4+ T cells, MALAT1 binds the PRC2 complex and removes H3K27 methylation at the HIV-1 LTR promoter, thereby activating viral transcription (132). LncRNA-SAF is significantly upregulated in HIV-1-infected human monocyte-derived macrophages and airway macrophages from HIV-1-infected individuals. Its elevated expression directly suppresses caspase-3/7 activity and blocks apoptosis in infected macrophages, thus sustaining persistent HIV-1 replication in these long-lived reservoir cells (133). Furthermore, HIV-1 infection induces the upregulation of lncRNA-uc002yug.2, which enhances HIV-1 replication and LTR promoter activity by downregulating RUNX1b and RUNX1c and upregulating Tat expression (the LTR promoter is a key regulator of HIV-1 transcription, and increased activity directly promotes viral replication) (134). Collectively, HIV-1 hijacks a diverse set of host ncRNAs in a cell- and target-specific manner to promote its own replication either directly or indirectly by blocking host antiviral innate immunity, antagonizing host restriction factors, regulating ceRNA networks, epigenetically activating viral transcription, and sustaining the survival of infected cells. This multilayered regulatory principle is highly similar to those of IAV and flaviviruses, revealing a conserved evolutionary strategy by which pathogenic RNA viruses exploit host ncRNAs for replication and survival, and provides new insights for targeting ncRNAs to eradicate chronic viral infections.

Collectively, this section outlines the conserved yet virus-specific strategies by which diverse pathogens hijack host ncRNA networks to remodel intracellular environments permissive for replication. Upon infection, most studied pathogens induce abundant proviral miRNAs, lncRNAs, and circRNAs; vtRNA proviral activity is uniquely characterized in IAV to date. These induced ncRNAs function through unified core mechanisms including suppressing RLR–IFN innate signaling, rewiring host metabolic and autophagic programs, stabilizing viral polymerase complexes, and encoding small functional peptides to sustain viral propagation. Despite shared reliance on ncRNA-mediated immune suppression across IAV, flaviviruses and HIV-1, clear distinctions exist in target selection and effector pathways: each pathogen recruits distinct ncRNA repertoires matched to its own replication cycle and cell tropism. Consistent with the four-category framework visualized in **Figures 1**, **3**, the widespread upregulation of proviral ncRNAs constitutes the primary viral tactic to subvert host defenses. A central takeaway from the above body of evidence is that viruses exploit the full spectrum of available ncRNAs in a coordinated, multi-layered manner rather than relying on a single ncRNA to establish favorable infection conditions.

**Figure 3:**
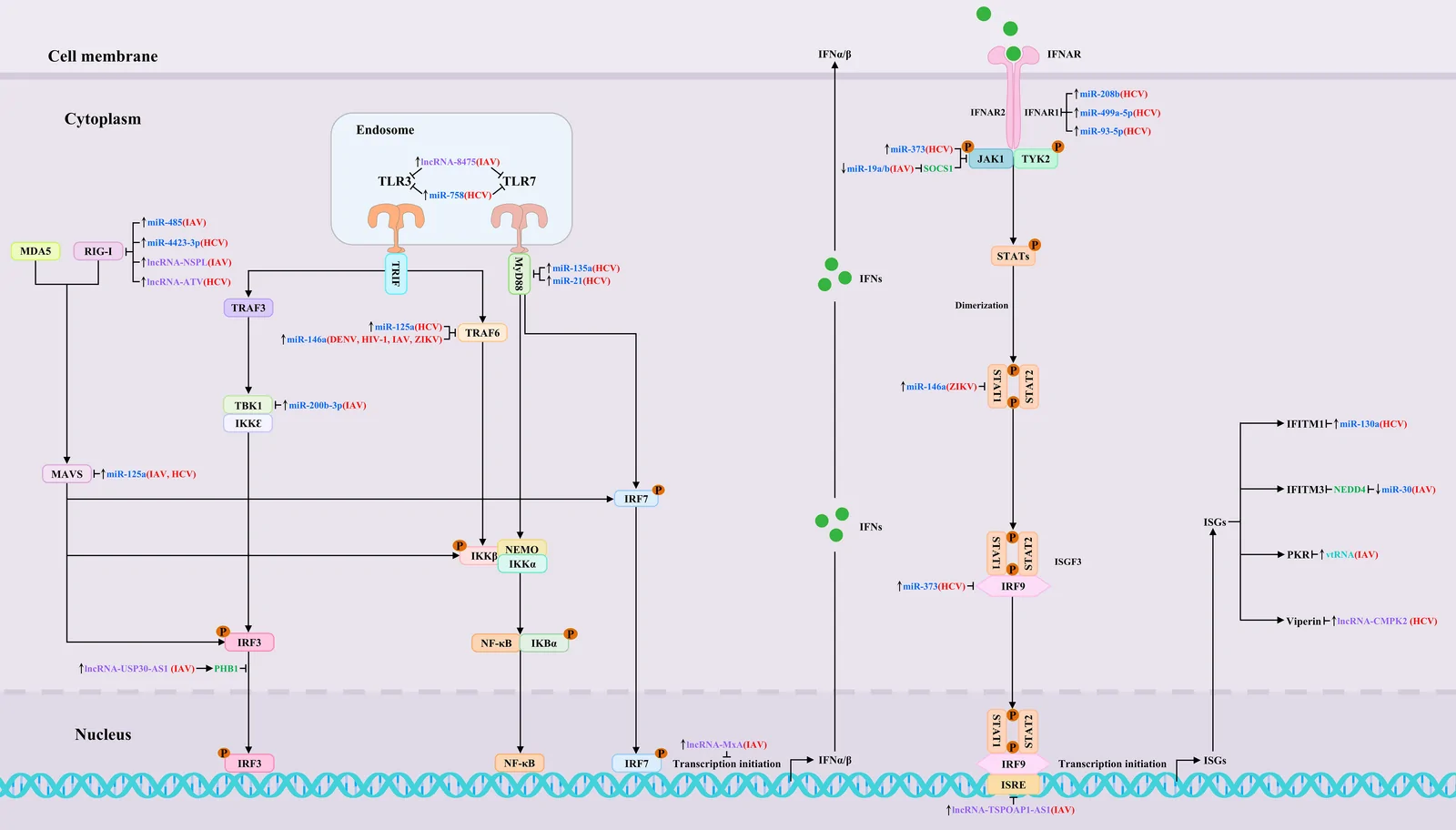
Viral manipulation of host ncRNA networks to subvert antiviral innate immune signaling pathways. To facilitate replication and immune evasion, viruses employ two core strategies by modulating host ncRNA expression: (1) upregulating proviral ncRNAs to hijack host machinery and block antiviral innate immune signaling; (2) downregulating antiviral ncRNAs to antagonize host defense and promote immune escape and efficient replication. These viral tactics remodel the intracellular microenvironment favorable for viral pathogenesis.

## 4 Viruses antagonize ncRNA-mediated host antiviral defense to enhance viral replication

IAV, including subtypes such as H5N1 and H1N1, has evolved sophisticated strategies to antagonize host ncRNA-mediated antiviral defense, regulating the expression of specific ncRNAs to promote its own replication and pathogenicity. Studies have demonstrated that multiple endogenous miRNAs exert antiviral effects by directly targeting distinct gene segments of the IAV genome: miR-324-5p and miR-491 both target the viral PB1 gene, miR-1249 and miR-584-5p co-target the viral PB2 gene, miR-1307-3p targets the viral NS1 gene, and miR-205-5p targets the viral NP gene (35, 135–138). These miRNAs bind to specific regions of their corresponding viral genes, thereby suppressing viral protein expression and IAV replication. Notably, IAV infection specifically downregulates the expression of these miRNAs, relieving their targeted inhibition of the respective viral genes, which in turn enhances the expression of viral proteins (e.g., PB1, PB2, NS1, NP) and ultimately facilitates viral replication. Furthermore, IAV infection downregulates a panel of endogenous miRNAs to relieve their targeted suppression of proviral host factors, leading to elevated expression of these factors and creating a favorable cellular environment for viral replication, assembly, and subsequent pathogenicity. A core subset of these miRNAs exerts antiviral effects by modulating conserved cell signaling pathways critical for viral replication: miR-193b directly binds the 3’UTR of β-catenin to potently inhibit Wnt/β-catenin signaling, inducing G0/G1 cell cycle arrest and delaying viral vRNP nuclear import to effectively suppress the replication of the H1N1 strain (139). miR-29a specifically targets FZD5, a key receptor of the Wnt-Ca²^+^ signaling pathway, to reduce endogenous FZD5 protein levels and diminish viral mRNA, protein levels, and progeny production across multiple IAV strains, with IAV infection downregulating both miRNAs to abrogate their inhibitory effects on viral replication via the Wnt pathway (140). Another major group of miRNAs exerts antiviral activity by activating IFN-related signaling cascades—the central host antiviral defense system—and IAV counteracts this by downregulating such miRNAs: miR-19a/b directly targets SOCS1 to abrogate its inhibitory effect on the IFN/JAK/STAT signaling pathway, thereby activating the host type I IFN-mediated antiviral immune response and suppressing IAV replication (141). miR-26a directly suppresses USP3, a negative regulator of type I IFN signaling, to effectively activate the transcription of type I IFN genes and the production of downstream ISGs (142). The miR-30 family exerts dual-targeting antiviral effects by directly targeting SOCS1 and SOCS3 to relieve their suppression of the IFN/JAK/STAT pathway, while also inhibiting NEDD4 (a negative regulator of IFITM3) to reinforce IFITM3-mediated defense against IAV entry and replication (143). miR-302a directly binds the 3’UTR of IRF5 to downregulate its expression, and IRF5 promotes viral replication, with IAV infection downregulating miR-302a to elevate IRF5 expression and enhance viral replication (144). miR-206 targets TNKS2 to activate the JNK/c-Jun signaling pathway, inducing type I IFN expression and enhancing STAT signal transduction, while miR-9–1 targets TNKS1 to promote type I IFN production and STAT1 phosphorylation, both strengthening the IFN-mediated antiviral response to inhibit viral replication, and adenovirus-mediated pulmonary delivery of either miRNA significantly reduces viral load in IAV-infected mice (145, 146). Besides, miR-126a-5p directly targets ADAMTS-4 mRNA to inhibit its expression, attenuating excessive inflammation, apoptosis, and matrix protease secretion in lung fibroblasts to suppress H1N1 replication, with IAV infection downregulating miR-126a-5p to promote ADAMTS-4 expression and reverse these effects (147). miR-1-3p is also downregulated upon IAV infection. It directly targets the 3’UTR of proviral host factor ATP6V1A to inhibit its expression, thereby reducing viral M1 mRNA and NP protein levels and suppressing viral replication, and knockdown of ATP6V1A can mimic its antiviral effect (148). miR-17-3p, also rapidly downregulated early in infection, directly targets GALNT3 mRNA to inhibit mucin-type O-glycosylation, with GALNT3 upregulated in an IAV replication-dependent manner post miR-17-3p downregulation to sustain viral replication (149). miR-18a-5p is significantly downregulated in a dose- and time-dependent manner in H5N1 infection models, directly targeting the 3’UTR of NEDD9 mRNA to inhibit H5N1 NP and M1 protein replication, and IAV infection downregulates miR-18a-5p to relieve NEDD9 inhibition and promote viral replication (150). Additionally, miR-221 exerts dual antiviral effects—directly targeting GALNT3 mRNA to inhibit mucin-type O-glycosylation and block IAV replication, while suppressing SOCS1 mRNA to activate the NF-κB signaling pathway and enhance IAV-triggered IFN-α/β and ISGs expression. IAV infection rapidly downregulates miR-221 in a dose- and time-dependent manner during the early stage, relieving its inhibition on viral replication to facilitate its own proliferation (149, 151). miR-33a directly binds the 3’UTR of ARCN1 to downregulate its expression, inhibiting viral replication by hindering ARCN1-mediated viral internalization and, independently, attenuating vRNP activity. IAV infection downregulates miR-33a to relieve this inhibitory effect and facilitate its own proliferation (152). IAV infection downregulates miR-548an as early as 3 hours post-infection in human alveolar and bronchial epithelial cells. miR-548an exerts its function by regulating NS1ABP expression. Overexpression of miR-548an decreases NS1ABP levels, promotes apoptosis of infected cells, and inhibits viral replication (153). miR-let-7b/f is significantly downregulated upon IAV infection, directly targeting the 3’UTR of RPS16 to downregulate its expression and upregulate type I IFN and downstream ISGs, thereby inhibiting viral replication, with IAV infection reducing miR-let-7b/f levels to relieve RPS16 inhibition and promote replication (154). Beyond miRNAs, lncRNAs also act as key antiviral effectors and are antagonized by IAV: LncRNA-LRIR is significantly downregulated in A549 cells post IAV infection, exerting antiviral activity via its 258–381 nt and 38–97 nt sequence regions to directly inhibit viral genome replication/transcription and downregulate host TMPRSS2 (a protease critical for viral entry), with IAV suppressing LRIR expression to relieve TMPRSS2 inhibition and facilitate viral replication (18). Taken together, these findings underscore the diverse and sophisticated ncRNA-mediated antiviral mechanisms employed by the host to restrict IAV replication, and how IAV has strategically evolved to downregulate these antiviral ncRNAs to dismantle host defense networks, highlighting the intricate and dynamic interplay between host and virus at the ncRNA level.

Similar to IAV, members of the Flavivirus genus, including DENV, HCV, and ZIKV, have also evolved sophisticated strategies to antagonize host ncRNA-mediated antiviral defense by downregulating the expression of specific ncRNAs, thereby promoting their own infection and replication. miR-133a can exert antiviral effects by targeting the 3’UTR of the RBMX gene to inhibit its expression and viral RNA levels, while DENV infection significantly downregulates this miRNA to relieve its inhibition on RBMX, thereby partially rescuing viral replication (155). Furthermore, DENV infection downregulates miR-155 in a time-dependent manner. Exogenous overexpression of this miRNA can limit viral replication by targeting Bach1, inducing HO-1-mediated inhibition of NS2B/NS3 protease activity and IFN response. Accordingly, DENV-induced downregulation of miR-155 relieves its antiviral effect, creating favorable conditions for its own proliferation (156). DENV infection downregulates miR-223 through the transcription factors C/EBPα and E2F1, and this miRNA can target the 3’UTR of STMN1 to reduce its expression and inhibit viral replication. This DENV-mediated miR-223 downregulation relieves the inhibition of STMN1 by miR-223, ultimately promoting DENV replication (157). With respect to HCV infection, it significantly downregulates the miR-130 family, miR-25, and miR-let-7. These miRNAs exert antiviral functions by inhibiting the expression of host co-factors essential for HCV replication. This regulatory process relieves their inhibition on host cofactors, promotes the expression of the latter, and further drives its own infection at multiple stages of the viral life cycle (158). Interestingly, HCV infection downregulates miR-130b and miR-185, both of which can independently inhibit HCV infection. Among them, miR-185 can also exert broad-spectrum antiviral effects by regulating host metabolic pathways, and both can inhibit host lipid accumulation. By downregulating these two miRNAs, HCV relieves their inhibition on lipid accumulation, promotes lipid synthesis, and further antagonizes the antiviral effect of 25-HC, facilitating its own replication (159). HCV infection downregulates miR-181c by regulating C/EBP-β. This miRNA can target HOXA1 and its downstream STAT3 and STAT5, and also bind to HCV E1 and NS5A sequences to inhibit viral replication, while this downregulation event effectively relieves its antiviral effect (160). miR-185-5p can target GALNT8 to inhibit HCV replication. HCV infection downregulates this miRNA, thereby reversing its antiviral effect by relieving its inhibition on GALNT8 (161). miR-200c can target OCLN to reduce its expression and inhibit HCV infectivity, and HCV infection downregulates this miRNA to relieve its inhibition on its own infection (162). For HCV (JFH-1 strain), its infection downregulates miR-29c, which can target STAT3 and inhibit viral replication by promoting type I IFN response. This downregulation trend of miR-29c relieves its inhibition on STAT3, thereby promoting HCV replication (163). HCV infection downregulates miR-451a, which can target GK to inhibit lipid accumulation and viral replication. This regulatory mode relieves the inhibition on GK, providing the necessary conditions for HCV replication through lipid accumulation (164). HCVcc infection downregulates miR-99a, which can inhibit HCV replication by inhibiting the mTOR/SREBP-1c pathway and reducing lipid accumulation. This downregulation process relieves the inhibition, facilitating viral replication and lipid accumulation (165). In addition to miRNAs, HCV infection also downregulates the lncRNA-Pint, which can interact with SRPK2 to inhibit lipogenesis and viral replication. The downregulation of Pint promotes lipogenesis, creating favorable conditions for its own replication and liver disease progression (166). For ZIKV, its infection downregulates miR-142-5p in human umbilical cord mesenchymal stem cells. This miRNA can target IL6ST and ITGAV to inhibit viral replication (ITGAV is involved in ZIKV entry), and this ZIKV-induced downregulation relieves its inhibitory effect, promoting its own replication in umbilical cord tissues (167). In summary, DENV, HCV, and ZIKV all specifically downregulate host miRNAs and lncRNAs to disrupt their mediated antiviral defense, regulate host cell metabolism and signaling pathways, and create favorable conditions for their own replication. These regulatory mechanisms not only reveal the pathogenic characteristics of flaviviruses but also provide potential targets for the development of new antiviral strategies.

In addition to IAV and members of the Flavivirus genus, HIV-1 and SARS-CoV-2 can also specifically regulate the expression of host ncRNAs to antagonize their mediated antiviral defense responses, thereby creating favorable conditions for their own replication, latency, and transmission. For HIV-1, as an important host antiviral miRNA, miR-191-5p is significantly downregulated in HIV-1-infected patients. It can inhibit the expression of CCR1 and NUP50 by targeting them, thereby exerting an anti-HIV-1 replication effect. HIV-1-induced downregulation of this miRNA relieves its antiviral inhibition, facilitating its own proliferation (168). LncRNA-GAS5 tends to be downregulated during HIV-1 infection. It can exert an anti-HIV-1 replication function by interacting with and inhibiting the activity of miR-873, and HIV-1-induced downregulation of GAS5 after infection relieves its inhibition on miR-873, indirectly promoting its own replication, which also provides potential targets for the antiviral treatment of HIV/AIDS (169). LncRNA-NKILA can inhibit HIV-1 LTR promoter activity and viral replication by suppressing the NF-κB signaling pathway and blocking the recruitment of p65 to the κB binding sites in HIV-1 LTR. Notably, HIV-1 infection or latency reactivation can significantly downregulate NKILA expression by reducing the acetylation level of histone K27 in the NKILA promoter region, relieving its inhibition on the NF-κB pathway and viral replication, while promoting the reactivation of latent HIV-1 (170). LncRNA-NRON can exert an anti-HIV-1 replication function by regulating the activity of the transcription factor NFAT, and HIV-1 downregulates the expression of NRON through the early accessory protein Nef, thereby enhancing NFAT activity and viral LTR function to promote its own replication and persistent infection (171). Turning to SARS-CoV-2 infection, a variety of host miRNAs can exert antiviral effects by targeting the viral genome, and all of them are specifically downregulated by the virus to relieve their inhibitory effects. miR-298, miR-508, miR-1909, and miR-3130 can directly bind to the RNA sequence of the SARS-CoV-2 S protein, inhibiting S protein expression and viral replication. SARS-CoV-2 infection significantly downregulates the expression of these four miRNAs, relieving their inhibition on the S protein and its own replication, and the targeting sequences of these miRNAs are conserved among SARS-CoV-2 variants of concern, suggesting their broad-spectrum antiviral potential (172). In addition, miR-150-5p is sharply downregulated in the plasma of patients with moderate-severe COVID-19. This miRNA can inhibit SARS-CoV-2 infection by directly binding to the miRNA recognition element (MRE) on the coding strand of SARS-CoV-2 Nsp10, thereby suppressing Nsp10 expression. SARS-CoV-2 reverses its antiviral effect by downregulating the expression of miR-150-5p, providing favorable conditions for its own infection (173). Notably, current research on SARS-CoV-2-modulated host ncRNAs remains preliminary and fragmented compared with IAV and HCV. Most available studies focus merely on microRNAs targeting viral structural proteins, while systematic regulatory networks governed by lncRNAs and circRNAs remain poorly characterized. Further high-throughput profiling and functional validation studies are required to fully delineate layered ncRNA-based host–virus crosstalk during COVID-19 infection. In summary, although HIV-1 and SARS-CoV-2 differ from flaviviruses in their pathogenic mechanisms, both disrupt host antiviral defense and regulate host signaling pathways as well as viral gene expression by downregulating host miRNAs and lncRNAs with antiviral functions. These mechanisms not only deepen the understanding of their pathogenic characteristics but also provide potential targets for the development of novel antiviral drugs, and together with the regulatory principles of IAV and members of the Flavivirus genus, they form the core mechanism by which viruses antagonize host ncRNA-mediated antiviral defense (174–177).

Collectively, this section details diverse antagonistic tactics deployed by representative human viruses to disrupt ncRNA-dependent host immune defense. A broad spectrum of antiviral miRNAs and lncRNAs are selectively suppressed post infection across IAV, flaviviruses, HIV-1 and SARS-CoV-2; antagonistic effects mediated by vtRNAs have not yet been documented in existing research. These downregulated ncRNAs converge on shared antiviral axes including RLR sensing, IFN cascade activation and host restriction factor function, while virus-specific regulatory differences exist in target transcripts and inhibitory efficiency. Many viruses repress ncRNAs that directly bind viral genomes or suppress viral polymerase activity, as well as those that amplify interferon signaling. Consistent with the four-category classification system illustrated in **Figures 1**, **3**, targeted silencing of antiviral ncRNAs constitutes a universal viral immune evasion mechanism. A central takeaway from the above body of evidence is that viruses antagonize host defense through widespread repression of antiviral ncRNAs, rather than relying on single inhibitory pathways to dismantle multilayered host antiviral programs.

## 5 NcRNA-mediated regulatory principles and future research directions of virus–host interactions in viral replication

In various viral infections, host ncRNA-mediated regulation follows four evolutionarily conserved and functionally important principles. First, ncRNAs can target conserved pathways to regulate the replication of various distinct viruses through identical molecular mechanisms. miR-146a is broadly upregulated during infection by IAV, DENV, ZIKV, and HIV-1 and inhibits IFN-β and downstream ISGs expression by targeting TRAF6, thereby facilitating viral replication (19–22). Similarly, miR-125a also exhibits cross-viral conserved regulatory principles. It is elevated in both IAV and HCV infections, suppresses IFN signaling via targeting MAVS, and weakens host antiviral defenses to promote viral propagation (23, 24). Second, the same ncRNA can mediate viral replication through entirely distinct regulatory principles depending on the type of virus. LncRNA-GAS5 is upregulated in IAV infection and promotes viral replication by encoding a micropeptide to activate the Wnt/β-catenin pathway (26). It is also increased during HCV infection and inhibits viral replication by binding the NS3 protein, whereas it is downregulated in HIV-1 infection to indirectly enhance viral replication by relieving repression of miR-873 (25, 169). miR-200c likewise displays virus-specific regulation: it targets CNTN1 to restore the RIG-I-MAVS antiviral axis in IAV infection while reducing HCV infectivity by decreasing OCLN expression, showing distinct directions and mechanisms between viruses (34, 162). Third, a single ncRNA can exert multiple layers of regulation on a single type of virus via multiple parallel pathways. miR-21-3p promotes IAV replication by separately regulating HDAC8 and FGF2 (72, 73). miR-221 exhibits dual antiviral effects. It directly targets GALNT3 mRNA to inhibit mucin-type O-glycosylation and block IAV replication, while repressing SOCS1 to activate NF-κB and IFN pathways and strengthen antiviral immunity (149, 151). The miR-30 family concurrently targets SOCS1/SOCS3 and NEDD4/IFITM3 to dually enhance anti-IAV responses (143). miR-373 comprehensively suppresses host antiviral immunity in HCV infection by simultaneously targeting JAK1/IRF9 and IRF5 (114, 115). LncRNA-MALAT1 is markedly upregulated in HIV-1-infected macrophages and CD4^+^ T cells and promotes viral replication in a cell-type-specific manner. In macrophages, it sponges miR-145-5p and miR-150-5p to suppress innate immunity, whereas in CD4^+^ T cells, it binds the PRC2 complex to activate viral transcription (130–132). Fourth, multiple distinct ncRNAs can converge to target the same host factor or pathway, thereby cooperatively and precisely regulating the replication of a single virus. During IAV infection, miR-17-3p and miR-221 jointly target GALNT3. They respectively inhibit mucin-type O-glycosylation and modulate replication-dependent protein levels to fine-tune viral replication (149). Additionally, IAV-induced lncRNA-9101 and lncRNA-LINC02574 both target MDA5 and positively regulate MDA5-mediated type I IFN response and downstream ISGs expression, thereby synergistically reinforcing host antiviral signaling (45, 50).

Beyond these conserved regulatory principles, host ncRNAs exhibit remarkable functional diversity and regulatory flexibility during viral infection. On one hand, numerous ncRNAs achieve integrated multifunctional control by simultaneously governing antiviral signaling cascades, viral replication cycles, and stress granule assembly. For example, circRNA-CBP exerts robust anti-IAV activity by binding viral NP to impair vRNP function, interacting with NS1 to restore IFN response, and associating with G3BP1 to facilitate stress granule formation (59). LncRNA-RPS6P3 binds viral NP to block vRNP assembly and targets NS1 to restore RIG-I signaling, consequently elevating IFN-β production and activating host antiviral immunity (53). On the other hand, certain lncRNAs achieve multilayered regulation through nucleocytoplasmic dual functions. LncRNA-AVAN promotes FOXO3a transcription in the nucleus and enhances RIG-I ubiquitination in the cytoplasm, thereby efficiently suppressing IAV replication (46). Furthermore, several ncRNAs form feedback loops with PRRs. RIG-I induces lncRNA-LINC02574 expression, which in turn maintains the levels of RIG-I, MDA5, and TLR3, amplifies IFN signaling, and establishes a positive feedback circuit to potentiate antiviral defense (50).

Based on the achievements accumulated in previous studies, drawing on the molecular mechanisms of viral replication regulation mediated by different host ncRNAs, further in-depth study of known regulatory mechanisms and exploration of unknown regulatory mechanisms are among the core future research trends in the field of host ncRNA-virus interactions. Although current research has established a relatively systematic theoretical framework for how host ncRNAs regulate viral replication, there are still obvious gaps in the research progress of regulatory mechanisms among different viruses. For instance, compared with other representative viruses, the research findings on the regulation of SARS-CoV-2 replication by host ncRNAs are still relatively scarce. Therefore, strengthening the cross-reference of research ideas on regulatory mechanisms related to different viruses is of great significance for filling this gap (1, 6, 9). Specifically, it is necessary to further screen and identify host ncRNAs involved in the regulation of SARS-CoV-2 replication and systematically decipher the molecular mechanisms through which they exert their effects. In addition, it remains an urgent scientific question to be answered whether the regulatory mechanisms of these ncRNAs are associated with proviral host factors, host restriction factors, other ncRNAs (via sponging mechanisms), micropeptides, lipids, autophagy, apoptosis, metabolic reprogramming, and stress granule formation. Taking the future research ideas on the regulatory mechanisms of SARS-CoV-2 as a reference, other viruses (including DENV, ZIKV, HIV-1, other known viruses, and future unknown emerging viruses) can further explore their host ncRNA-related regulatory mechanisms in combination with their own existing research progress and based on a comprehensive understanding of conserved regulatory principles and functional diversity of ncRNAs in host-virus interactions. Importantly, some studies have not yet clarified the specific viral proteins or host pathways that mediate the regulation of host ncRNA expression. Moreover, elucidating the molecular mechanisms by which different ncRNAs regulate viral replication at the single-cell level still faces many challenges. These directions are all worthy of in-depth research, which can not only deepen our understanding of the regulatory mechanisms of host ncRNA-virus interactions but also provide an important theoretical basis for the future development of new antiviral drugs targeting these host ncRNAs and their regulatory mechanisms.

Notably, although significant progress has been made in elucidating the molecular mechanisms by which host ncRNAs regulate viral replication in recent years, several critical knowledge gaps remain to be addressed. Taking IAV as a representative example, there are still a number of ncRNAs that have been confirmed to be involved in regulating viral replication but lack in-depth mechanistic characterization, including lncRNA-ISR, lncRNA-PSMB8-AS1, and lncRNA-VIN (178–180). Meanwhile, whether circRNAs, like lncRNAs, can influence viral replication by encoding or regulating micropeptides remains to be clarified (26, 96, 100). Additionally, it is also unclear whether IAV antagonizes innate immunity and promotes its own replication by impairing circRNA-mediated host antiviral effects (18). Furthermore, whether the regulation of IAV replication by ncRNAs is associated with the cGAS-STING signaling pathway remains a key direction for future investigation. In-depth dissection of these unresolved questions will further refine the theoretical framework of host ncRNA-virus interactions centered on viral replication regulation.

## 6 Concluding remarks and future perspectives

Collectively, host ncRNAs act as master regulators of host antiviral defense and viral replication in virus–host interactions. As outlined in **Figure 1**, these multifaceted host ncRNA–virus interactions can be classified into four regulatory subgroups defined by ncRNA biological functions and infection-associated expression dynamics. The host establishes a multilayered antiviral defense system by upregulating antiviral ncRNAs or downregulating proviral ncRNAs. In turn, major pathogens threatening global public health, including IAV, DENV, HCV, ZIKV, HIV-1, and SARS-CoV-2, hijack or antagonize host ncRNA networks to reshape the intracellular milieu and establish a replication permissive state for efficient propagation. This review systematically summarizes the regulatory principles of ncRNAs in governing viral replication and highlights their multifaceted regulatory roles in antiviral innate immune signaling pathways, viral replication cycles, autophagy, apoptosis, metabolic reprogramming, and stress granule formation. The regulatory mechanisms by which differentially expressed ncRNAs modulate host antiviral innate immune signaling pathways upon viral infection are illustrated in **Figures 2**-**4**. The roles of diverse ncRNAs implicated in modulating viral replication are summarized in **Tables 1**–**4**. In-depth dissection of these host ncRNA–virus interactive regulatory principles will further refine the theoretical framework of host ncRNA-virus interactions centered on viral replication regulation (1, 9).

**Figure 4:**
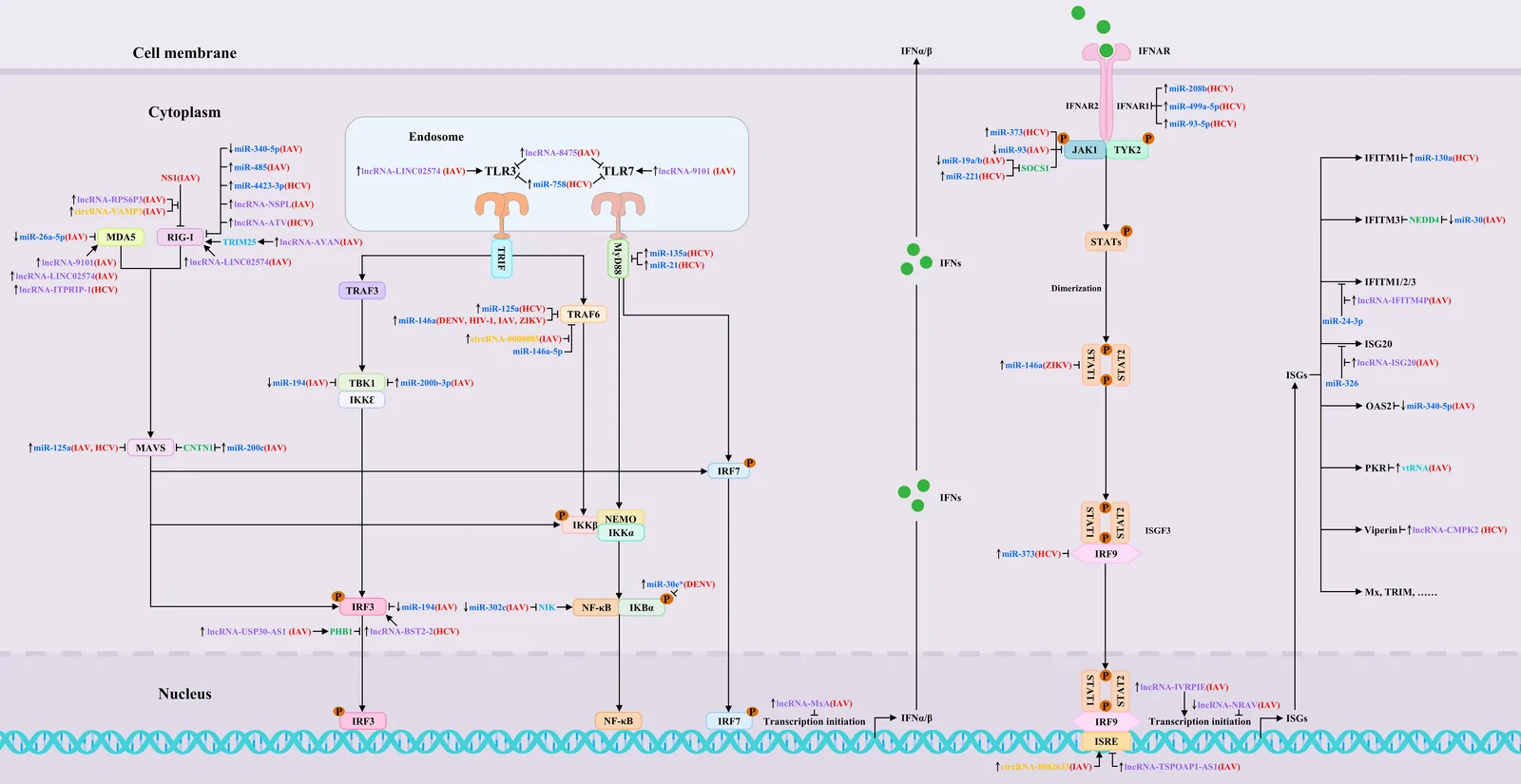
Host antiviral innate immune signaling pathways regulated by ncRNAs. PRRs recognize viral RNA and activate downstream innate immune signaling cascades, thereby inducing the expression of IFNs and ISGs to restrict viral replication. Host ncRNAs, including miRNAs, lncRNAs, circRNAs, and vtRNAs, directly or indirectly modulate multiple steps of these signaling pathways, from PRRs activation to interferon induction and ISGs expression, thereby fine-tuning host antiviral responses and viral replication. The red, blue, purple, orange, peacock blue, green, and light blue colors, respectively, represent viral proteins (viruses), miRNAs, lncRNAs, circRNAs, vtRNAs, proviral host factors, and host restriction factors.

**Table 1:** Roles of microRNAs implicated in modulating IAV replication.

MicroRNAs	Targets	Effect on IAV replication	Expression level after IAV infection	IAV subtypes	Cell lines	Ref.
miR-101	mTOR, ↓	Inhibitive	↑	a reassortant between H1N1 and H3N2	A549	(37)
miR-1249	PB2, ↓	Inhibitive	↓	H5N1	A549	(136)
miR-125a/b	MAVS, ↓	Promotive	↑	H1N1, H3N2	pBECs	(23)
miR-126a-5p	ADAMTS-4, ↓	Inhibitive	↓	H1N1	HFL-1	(147)
miR-1290	VIM, ↓	Promotive	↑	H1N1	A549	(85)
miR-1307-3p	NS1, ↓	Inhibitive	↓	H1N1	A549	(137)
miR-132-3p	IRF1, ↓	Promotive	↑	H1N1	A549	(86)
miR-1-3p	ATP6V1A, ↓	Inhibitive	↓	H1N1, H3N2	A549	(148)
miR-146a	TRAF6, ↓	Promotive	↑	H1N1	A549	(19)
miR-17-3p	GALNT3, ↓	Inhibitive	↓	H1N1, H3N2	A549	(149)
miR-18a-5p	NEDD9, ↓	Inhibitive	↓	H5N1	A549	(150)
miR-19a/b	SOCS1, ↓	Inhibitive	↓	H1N1	A549	(141)
miR-193b	β-catenin, ↓	Inhibitive	↓	H1N1	A549, HEK293	(139)
miR-193b-5p	occludin, ↓	Promotive	↑	H1N1	BEAS2b	(87)
miR-194	Phosphorylation of TBK1 and IRF3, ↓ FGF2, ↓	Promotive	↓	H1N1	A549, HEK293T	(71)
miR-200b-3p	TBK1, ↓	Promotive	↑	H1N1	HEK293T	(88)
miR-200c	CNTN1, ↓	Inhibitive	↑	H5N6	A549	(34)
miR-203	DR1, ↓	Inhibitive	↑	H5N1	A549	(38)
miR-205-5p	NP, ↓	Inhibitive	↓	H1N1	MLE-12	(138)
miR-206	TNKS2, ↓	Inhibitive	↓	H1N1	HEK293T	(145)
miR-21-3p	HDAC8, ↓	Promotive	↓	H1N1, H5N1	A549	(72)
miR-21-3p	FGF2, ↓	Promotive	↓	H5N1	A549	(73)
miR-221	GALNT3, ↓	Inhibitive	↓	H1N1, H3N2	A549	(149)
miR-221	SOCS1, ↓	Inhibitive	↓	H1N1	A549	(151)
miR-26a	USP3, ↓	Inhibitive	↓	H1N1	A549, HEK293T	(142)
miR-26a-5p	MDA5, ↓	Promotive	↓	H5N1	HD11	(74)
miR-29a	FZD5, ↓	Inhibitive	↓	H1N1	HEK293T	(140)
miR-30	SOCS1,SOCS3, NEDD4, ↓	Inhibitive	↓	H5N1	A549	(143)
miR-302a	IRF5, ↓	Inhibitive	↓	H1N1	A549, PBMC	(144)
miR-302c	NIK, ↓	Promotive	↓	H3N2	A549	(75)
miR-323	PB1, ↓	Inhibitive	↑	H1N1	MDCK, HEK293T	(35)
miR-324-5p	PB1, ↓	Inhibitive	↓	H1N1, H5N1	A549, SAEC	(135)
miR-33a	ARCN1, ↓	Inhibitive	↓	H1N1, H3N2, H9N2	A549, HEK293T, Hela	(152)
miR-340-5p	RIG-I, ↓ OAS2, ↓	Promotive	↓	H5N1	A549	(76)
miR-34c	PLK4, ↑	Promotive	↑	H1N1	A549	(89)
miR-4276	COX6C, ↓	Promotive	↓	H1N1, H3N2	A549	(77)
miR-4776	NFKBIB, ↓	Promotive	↑	H1N1	HBEpC	(90)
miR-485	RIG-I, ↓	Promotive	↑	H5N1	HEK293T	(181)
	PB1, ↓	Inhibitive	↑	H5N1	HEK293T	(181)
miR-491	PB1, ↓	Inhibitive	↓	H1N1	MDCK, HEK293T	(35)
miR-548an	NS1ABP, ↓	Inhibitive	↓	H1N1	A549	(153)
miR-584-5p	PB2, ↓	Inhibitive	↓	H5N1	A549	(136)
miR-654	PB1, ↓	Inhibitive	↑	H1N1	MDCK, HEK293T	(35)
miR-664a-3p	LIF, ↓	Promotive	↑	H3N2, H7N9	A549	(91)
miR-9	MCPIP1, ↓	Promotive	↑	H1N1, H3N2	A549	(92)
miR-9-1	TNKS1, ↓	Inhibitive	↓	H1N1	HEK293T	(146)
miR-93	JAK1, ↓	Promotive	↓	H1N1	AT2, MLE-12	(78)
miR-let-7b/f	RPS16, ↓	Inhibitive	↓	H1N1	A549	(154)
miR-let-7c	M1, ↓	Inhibitive	↑	H1N1	A549	(36)

Column Expression level after viral infection: ↑: non‑coding RNA expression is upregulated upon viral infection, ↓: non‑coding RNA expression is downregulated upon viral infection.

Targets Column: ↓indicates that the corresponding ncRNA suppresses/down‑regulates the listed target gene or viral gene.

**Table 2:** Roles of lncRNAs, circRNAs, and vtRNAs implicated in modulating IAV replication.

LncRNAs/CircRNAs	Effect on IAV replication	Infection expression	Mechanisms	Ref.
lncRNA-01615	Inhibitive	↑	Elevates IFN-β, IL-28A, IL-29 and subsequent ISGs	(43)
lncRNA-45	Inhibitive	↑	Inhibits viral polymerase activity and NP/PA nuclear accumulation	(39)
lncRNA-61	Inhibitive	↑	Inhibits viral polymerase activity and NP/PA nuclear accumulation	(40)
lncRNA-155	Inhibitive	↑	Inhibits PTP1B to relieve type I IFN signaling suppression	(44)
lncRNA-8475	Promotive	↑	Inhibits TLR3/TLR7 signaling to block IFN and ISGs expression	(45)
lncRNA-9101	Inhibitive	↑	Activates TLR7/MDA5 signaling to boost IRF7 and IFN-β transcription	(45)
lncRNA-ACOD1	Promotive	↑	Binds GOT2 to enhance catalytic activity, regulates metabolic networks	(93)
lncRNA-ALOX12	Promotive	↑	Binds PB2 to facilitate its nuclear import and viral RNA synthesis	(27)
lncRNA-AROD	Promotive	↓	Sequesters miR-324-5p to upregulate CUEDC2 and repress IFN-β/ISGs	(79)
lncRNA-AVAN	Inhibitive	↑	Binds TRIM25, enhances RIG-I ubiquitination to promote IFN expression	(46)
lncRNA-GAS5	Promotive	↑	Modulates NOTUM via micropeptide to activate Wnt/β-catenin	(26)
lncRNA-GBP1P1	Inhibitive	↑	Sequesters DHX9 to restrict viral mRNA utilization	(41)
lncRNA-IFITM4P	Inhibitive	↑	Sponges miR-24-3p to stabilize IFITM1/2/3 transcripts	(47)
lncRNA-IPAN	Promotive	↑	Binds IAV PB1 to stabilize it and block RIG-I-TRIM25-mediated degradation	(94, 95)
lncRNA-ISG20	Inhibitive	↑	Sponges miR-326 to relieve ISG20 translational repression	(48)
lncRNA-ISR	Inhibitive	↑	Not determined	(178)
lncRNA-IVRPIE	Inhibitive	↑	Binds hnRNP U to regulate histone modifications and ISGs transcription	(49)
lncRNA-LINC01197	Inhibitive	↑	Binds cytoplasmic PABPC1 to block viral mRNA translation efficiently	(42)
lncRNA-LINC02574	Inhibitive	↑	Maintains RIG-I/TLR3/MDA5 and enhances IFN responses	(50)
lncRNA-LRIR	Inhibitive	↓	Inhibits viral genome replication and negatively regulates TMPRSS2	(18)
lncRNA-MALAT1	Promotive	↑	Suppresses innate immunity via micropeptide to boost viral replication	(96)
lncRNA-MxA	Promotive	↑	Forms IFN-β promoter RNA-DNA triplex, suppresses RLR antiviral pathway	(97)
lncRNA-NEAT1	Inhibitive	↑	Boosts TBK1 activation to strengthen antiviral responses	(51)
lncRNA-NRAV	Promotive	↓	Modulates epigenetic ISGs transcription to restrain antiviral immunity	(16)
lncRNA-NSPL	Promotive	↑	Blocks TRIM25-mediated RIG-I ubiquitination to suppress antiviral signaling	(98)
lncRNA-PAAN	Promotive	↑	Binds PA to facilitate RdRp assembly and boost viral RNA synthesis	(99)
lncRNA-PCBP1-AS1	Promotive	↑	Enhances autophagy via micropeptide to facilitate viral proliferation	(100)
lncRNA-PINK1‐2:5	Inhibitive	↑	Upregulates TXNIP to exert broad anti-IAV restriction effects	(55)
lncRNA-PSMB8-AS1	Promotive	↑	Not determined	(179)
lncRNA-RDUR	Inhibitive	↑	Binds ILF2/ILF3 to boost IRF3 phosphorylation and induce antiviral ISGs	(52)
lncRNA-RPS6P3	Inhibitive	↑	Binds NP to suppress vRNP activity and binds NS1 to relieve RIG-I inhibition	(53)
lncRNA-SAAL	Inhibitive	↑	Upregulates Serpina3i to activate IFN-β and downstream ISGs	(54)
lncRNA-THRIL	Promotive	↓	Restrains IRF3 activation to block core IFN immune responses	(80)
lncRNA-TSPOAP1-AS1	Promotive	↑	Suppresses ISRE activation to reduce ISGs expression	(101)
lncRNA-up4	Promotive	↑	Suppresses IFN-β/Mx1/OAS1 to promote viral replication	(28)
lncRNA-USP30-AS1	Promotive	↑	Binds PHB1 to stabilize its protein and block IRF3 nuclear import	(102)
lncRNA-VIN	Promotive	↑	Not determined	(180)
circRNA-0008085	Inhibitive	↑	Sponges miR-146a-5p to upregulate TRAF6 and inhibit viral replication	(56)
circRNA-0050463	Promotive	↑	Sponges miR-33b-5p to upregulate EEF1A1 and facilitate viral replication	(103)
circRNA-0082633	Inhibitive	↑	Enhances ISRE activity and IFN-β to activate IFN signaling	(57)
circRNA-AIVR	Inhibitive	↑	Sponges miRNAs to upregulate CREBBP and promote IFN-β expression	(58)
circRNA-GATAD2A	Promotive	↑	Interacts with VPS34 to inhibit autophagy and promote viral replication	(104)
circRNA-CBP	Inhibitive	↑	Binds NP/NS1/G3BP1 to block vRNP and boost antiviral immunity	(59)
circRNA-MerTK	Promotive	↑	Suppresses the expression of type I IFNs and ISGs to impair host immunity	(105)
circRNA-MYO9A	Inhibitive	↑	Sponges miR-6059-3p to upregulate PAI-1 and reduce infectivity	(60)
circRNA-VAMP3	Inhibitive	↑	Binds NP/NS1 to impair vRNP activity and restore IFN-β activation	(61)
vtRNAs	Promotive	↑	Represses PKR activation to block IFN response and boost viral replication	(106)

↑: non‑coding RNA expression is upregulated upon viral infection, ↓: non‑coding RNA expression is downregulated upon viral infection.

**Table 3:** Roles of microRNAs implicated in modulating the replication of other viruses.

Viruses	MicroRNAs	Targets	Effect on viral replication	Expression level after viral infection	Ref.
DENV	miR-133a	RBMX, ↓	Inhibitive	↓	(155)
	miR-146a	TRAF6, ↓	Promotive	↑	(20)
	miR-155	Bach1, ↓	Inhibitive	↓	(156)
	miR-223	STMN1, ↓	Inhibitive	↓	(157)
	miR-30e*	IkBα, ↓	Inhibitive	↑	(62)
	miR-378	GrzB, ↓	Promotive	↓	(81)
	miR-548g-3p	the stem loop A promoter element within the 5’UTR	Inhibitive	↑	(63)
HCV	miR-1225-3p	GAB3, ↓	Promotive	↓	(82)
	miR-125a	MAVS, ↓ TRAF6, ↓	Promotive	↑	(24)
	miR-125b-5p	HuR, ↓	Inhibitive	↑	(64)
	miR-130 miR-25 miR-let-7	N/A	Inhibitive	↓	(158)
	miR-130a	IFITM1, ↓	Promotive	↑	(108)
	miR-130b miR-185	N/A	Inhibitive	↓	(159)
	miR-135a	MyD88, ↓	Promotive	↑	(109)
	miR-181c	HOXA1, ↓	Inhibitive	↓	(160)
	miR-185-5p	GALNT8, ↓	Inhibitive	↓	(161)
	miR-200c	OCLN, ↓	Inhibitive	↓	(162)
	miR-208b miR-499a-5p	IFNAR1, ↓	Promotive	↑	(110)
	miR-21	MyD88, ↓	Promotive	↑	(111)
	miR-21-5p	PTEN, ↓	Promotive	↑	(112)
	miR-215	TRIM22, ↓	Promotive	↑	(113)
	miR-221	SOCS1, ↓ SOCS3, ↓	Inhibitive	↑	(65)
	miR-27a	ABCA1, ↓ RXRα, ↓	Inhibitive	↑	(66)
	miR-29c	STAT3, ↓	Inhibitive	↓	(163)
	miR-373	IRF9, ↓ JAK1, ↓	Promotive	↑	(114)
	miR-373	IRF5, ↓	Promotive	↑	(115)
	miR-4423-3p	RIG-I, ↓	Promotive	↑	(116)
	miR-451a	GK, ↓	Inhibitive	↓	(164)
	miR-758	TLR3/7, ↓	Promotive	↑	(117)
	miR-93-5p	IFNAR1, ↓	Promotive	↑	(118)
	miR-99a	mTOR, ↓	Inhibitive	↓	(165)
HIV-1	miR-146a	TRAF6, ↓	Promotive	↑	(22)
	miR-186 miR-210 miR-222	Dicer1, ↓ HIV-EP2, ↓ HRB, ↓	Inhibitive	↑	(69)
	miR-191-5p	NUP50, ↓	Inhibitive	↓	(168)
	miR-197-3p	DDX52, ↓	Promotive	↑	(126)
	miR-198	Cyclin T1, ↓	Inhibitive	↑	(70)
	miR-25/93	MARCH1, ↓	Promotive	↑	(127)
	miR-34c-5p	KAT2B/PCAF, ↓	Promotive	↓	(83)
	miR-422a	MECP2, ↓	Promotive	↑	(128)
SARS-CoV-2	miR-1909	S, ↓	Inhibitive	↓	(172)
	miR-298				
	miR-3130				
	miR-508				
	miR-150-5p	Nsp10, ↓	Inhibitive	↓	(173)
ZIKV	miR-103a-3p	OTUD4, ↓	Promotive	↑	(124)
	miR-142-5p	IL6ST, ↓ ITGAV, ↓	Inhibitive	↓	(167)
	miR-146a	TRAF6, ↓ STAT1, ↓	Promotive	↑	(21)

In the column Expression level after viral infection: ↑: non‑coding RNA expression is upregulated upon viral infection, ↓: non‑coding RNA expression is downregulated upon viral infection. In the Targets column: ↓indicates that the corresponding ncRNA suppresses/down‑regulates the listed target gene or viral gene.

**Table 4:** Roles of lncRNAs and circRNAs implicated in modulating replication of other viruses.

Viruses	LncRNAs/CircRNAs	Effect on viral replication	Infection expression	Mechanisms	Ref.
DENV	lncRNA-SUN2-AS1	Promotive	↑	Represses ISGs transcription to impair host immunity	(107)
HCV	lncRNA-ATV	Promotive	↑	Binds RIG-I to block innate signaling and facilitate HCV replication	(119)
	lncRNA-BST2-2	Inhibitive	↑	Binds IRF3 to promote its activation and suppress viral replication	(15)
	lncRNA-CMPK2	Promotive	↑	Represses ISGs expression to impair immunity	(17)
	lncRNA-EGOT	Promotive	↑	Suppresses ISGs activation to facilitate HCV replication	(120)
	lncRNA-GAS5	Inhibitive	↑	Binds HCV NS3 to inhibit its function and restrict viral replication	(25)
	lncRNA-HULC	Promotive	↑	Modulates viral IRES translation to facilitate HCV replication	(121)
	lncRNA-ITM2C-1	Promotive	↑	Upregulates GPR55 to repress ISGs and facilitate HCV replication	(122)
	lncRNA-ITPRIP-1	Inhibitive	↑	Binds MDA5 to enhance IFN signaling and restrict viral replication	(67)
	lncRNA-Pint	Inhibitive	↓	Interacts with SRPK2 to suppress lipogenesis and viral replication	(166)
	lncRNA-UCA1	Promotive	↑	Sponges miR-145-5p to elevate SOCS7 and inhibit IFN response	(123)
HIV-1	lncRNA-GAS5	Inhibitive	↓	Inhibits miR-873 activity to suppress HIV-1 replication	(169)
	lncRNA-HEAL	Promotive	↑	Binds FUS to enhance HIV promoter activity and CDK2 expression	(129)
	lncRNA-MALAT1	Promotive	↑	Sponges miRNAs to upregulate CHCHD2/SOCS1 and suppress immunity in macrophages; binds PRC2 to modify LTR histone and activate HIV transcription in CD4^+^ T cells	(130–132)
	lncRNA-NKILA	Inhibitive	↓	Inhibits NF-κB to block p65 recruitment and halt HIV-1 replication	(170)
	lncRNA-NRON	Inhibitive	↓	Regulates NFAT activity to suppress HIV-1 LTR function	(171)
	lncRNA-SAF	Promotive	↑	Suppresses caspase3/7 to block apoptosis and sustain replication	(133)
	lncRNA-uc002yug.2	Promotive	↑	Represses RUNX1b/c to elevate Tat and enhance HIV LTR activity	(134)
ZIKV	lncRNA-LINC08148	Promotive	↑	Binds SP1 to activate Src and facilitate ZIKV entry and replication	(125)
	lncRNA-OASL-IT1	Inhibitive	↑	Elevates IFN signaling to strengthen host antiviral defense	(68)
	lncRNA-SUN2-AS1	Promotive	↑	Represses ISGs transcription to impair host immunity	(107)
	circRNA-0007321	Promotive	↓	Modulates miR-492/NFKBID/NF-κB cascade	(84)

↑: non‑coding RNA expression is upregulated upon viral infection, ↓: non‑coding RNA expression is downregulated upon viral infection.

Although host ncRNAs hold great promise as antiviral targets, their context-dependent and highly versatile regulatory properties must be carefully considered during clinical translation. For instance, miR-485 exerts diametrically opposing effects on IAV replication in a viral dose-dependent manner, suppressing host immune responses and facilitating viral replication at low viral loads while directly targeting viral components to restrict viral proliferation at high viral loads, thereby dynamically switching between immune inhibition and viral containment (181). Additionally, miR-302 mediates species-specific differential regulatory outcomes, predominantly acting as an antiviral effector in mammalian hosts yet exerting proviral effects in non-mammalian systems such as avian hosts, leading to distinct impacts on viral replication in different host environments (182). Furthermore, lncRNA-DFRV generates two transcript isoforms with opposing functions. The long isoform suppresses viral replication by activating host defense pathways, whereas the short isoform facilitates viral propagation by attenuating immune responses (183). In brief, these examples illustrate the highly complex and context-dependent regulatory landscape of ncRNAs in virus-host interactions, highlighting the need to comprehensively evaluate their functional diversity when developing antiviral strategies to minimize potential off-target effects and adverse risks.

Upon this foundation, to meet the future prevention and control demands posed by unknown emerging viruses, the interactive regulatory principles between host ncRNAs and viruses still require further exploration and in-depth investigation. Undoubtedly, when confronted with unknown emerging viruses in the future, comprehensive dissection of the antiviral defense mechanisms mediated by host ncRNAs, as well as the strategies by which viruses hijack and antagonize host ncRNA regulatory networks to achieve immune evasion, will be critical for fully elucidating the pathogenic mechanisms of viral infections. Based on the regulatory principles summarized herein, whether diverse classes of ncRNAs can ultimately modulate emerging viral replication by targeting proviral host factors, host restriction factors, other ncRNAs (molecular sponge mechanisms), micropeptides, and lipids, thereby regulating a series of biological processes including antiviral innate immune signaling pathways, viral replication cycles, autophagy, apoptosis, metabolic reprogramming, and stress granule formation, represents a key scientific question that urgently needs to be addressed in future research. Overall, the regulatory mechanisms systematically reviewed herein will provide important references for the mechanistic study of the interactions between host ncRNAs and unknown emerging viruses, and will also lay a solid theoretical foundation for addressing public health security threats caused by unknown emerging viruses and developing novel antiviral strategies.

## Glossary

ABCA1: ATP-binding cassette subfamily A member 1
ADAMTS-4: A disintegrin and metalloproteinase with thrombospondin motifs 4
AIDS: Acquired immunodeficiency syndrome
AP-1: Activator protein 1
ARCN1: Archain 1
ATP6V1A: ATPase catalytic subunit A
Bach1: BTB and CNC homology 1
Caspases: Cysteine-dependent aspartate-directed proteases
CCR1: C-C chemokine receptor type 1
CDK2: Cyclin-dependent kinase 2
C/EBPα: CCAAT/enhancer-binding protein alpha
C/EBP-β: CCAAT/enhancer-binding protein beta
ceRNA: Competing endogenous RNA
c-Fos: Cellular Fos
cGAS: Cyclic GMP-AMP synthase
CHCHD2: Coiled-coil-helix-coiled-coil-helix domain containing 2
CircRNAs: Circular RNAs
c-Jun: Cellular Jun
CNTN1: Contactin 1
COVID-19: Coronavirus disease 2019
COX6C: Cytochrome c oxidase subunit 6C
CREB: cAMP response element-binding protein
CREBBP: CREB-binding protein
CUEDC2: CUE domain containing 2
DDX52: DEAD-box helicase 52
DENV: Dengue virus
DHX9: DEAH-box helicase 9
DNMT1: DNA methyltransferase 1
DR1: Down-regulator of transcription 1
E2F1: E2F transcription factor 1
EEF1A1: Eukaryotic translation elongation factor 1 alpha 1
ERK: Extracellular signal-regulated kinase
FGF2: Fibroblast growth factor 2
FOXO3a: Forkhead box O3a
FUS: Fused in sarcoma
FZD5: Frizzled 5
G3BP1: GTPase-activating protein SH3 domain-binding protein 1
GAB3: GRB2-associated binding protein 3
GALNT3/8: GalNAc transferase 3/8
GOT2: Glutamic-oxaloacetic transaminase 2
GPR55: G protein-coupled receptor 55
GrzB: Granzyme B
HCV: Hepatitis C virus
HDACs: Histonedeacetylases
HIV-1: Human immunodeficiency virus type 1
HIV-EP2: HIV enhancer-binding protein 2
hnRNP U: Heterogeneous nuclear ribonucleoprotein U
HO-1: Heme oxygenase 1
HOXA1: Homeobox A1
HRB: HIV Rev-binding protein
HSP90AA1: Heat shock protein 90 alpha family class A member 1
HuR: Human antigen R
IAV: Influenza A virus
IFIT1: Interferon-induced protein with tetratricopeptide repeats 1
IFITMs: Interferon-induced transmembrane proteins
IFNs: Interferons
IFNAR1: Interferon-α/β receptor 1
IFNL2/3: Interferon lambda 2/3
IFN-β: Interferon β
ILs: Interleukins
IL6ST: Interleukin 6 signal transducer
ILF2/3: Interleukin enhancer binding factor 2/3
IPS-1: IFN-β promoter stimulator 1
IRAK1: Interleukin-1 receptor-associated kinase 1
IRFs: Interferon regulatory factors
ISGs: IFN-stimulated genes
ISGF3: IFN-stimulated gene factor 3
ISRE: IFN-stimulated response elements
ITGAV: Integrin subunit alpha V
IκBα: The alpha inhibitor of NF-κB
JAK1: Janus kinase 1
JNK: c-Jun N-terminal kinase
KAT2B: Lysine acetyltransferase 2B
LC3: Microtubule-associated protein light chain 3
LIF: Leukemia inhibitory factor
LncRNAs: Long non-coding RNAs
LTR: Long terminal repeat
MARCH1: Membrane-associated RING-CH-type finger 1
MAVS: Mitochondrial antiviral signaling
MCPIP1: Monocyte chemoattractant protein-induced protein 1
MDA5: Melanoma differentiation-associated gene 5
MECP2: Methyl CpG binding protein 2
miRNAs: MicroRNAs
MRE: miRNA recognition element
mTOR: Mammalian target of rapamycin
Mx1: Myxovirus resistance 1
MyD88: Myeloid differentiation factor 88
NcRNAs: Noncoding RNAs
NEDD4/9: Neuronal precursor cell expressed developmentally downregulated 4/9
NFAT: Nuclear factor of activated T cells
NF-κB: Nuclear factor κappa-light-chain-enhancer of activated B cells
NFKBIB: NF-κB inhibitor beta
NFKBID: NF-κB inhibitor delta
NIK: NF-κB inducing kinase
NOTUM: Notum, palmitoleoyl-protein carboxylesterase
NS1ABP: Non-structural-1A binding protein
NUP50: Nucleoporin 50
OAS1/2: 2’-5’-oligoadenylate synthase 1/2
OCLN: Occludin
OTUD4: OTU deubiquitinase 4
PABPC1: Poly(A) binding protein cytoplasmic 1
PHB1: Prohibitin 1
PKCϵ: Protein kinase C-ϵ
PKR: Protein kinase R
PLK4: Polo-like kinase 4
PRC2: Polycomb repressive complex 2
PRRs: Pattern recognition receptors
PTEN: Phosphatase and tensin homolog
PTP1B: Protein tyrosine phosphatase 1B
RBMX: RNA binding motif protein X-linked
RdRp: RNA-dependent RNA polymerase
RIG-I: Retinoic acid-inducible gene-I
RLR: RIG-I like receptor
RPS16: Ribosomal protein S16
RUNX1b/1c: Runt-related transcription factor 1b/1c
RXRα: Retinoid X receptor alpha
SARS-CoV-2: Severe acute respiratory syndrome coronavirus 2
SERPINE1: Serpin family E member 1
SOCS1/3/7: Suppressors of cytokine signaling 1/3/7
Src: SRC proto-oncogene non-receptor tyrosine kinase
SREBP-1c: Sterol regulatory element-binding protein 1c
SRPK2: Serine/arginine-rich protein kinase 2
STATs: Signal transducers and activators of transcriptions
STING: Stimulator of interferon genes
STMN1: Stathmin 1
TBK1: TANK-binding kinase 1
TCR: T cell receptor
TLR3/7: Toll-like receptor 3/7
TMPRSS2: Transmembrane protease serine 2
TNKS1/2: Tankyrase 1/2
TRAF6: TNF receptor-associated factor 6
TRIM25: Tripartite motif protein 25
TXNIP: Thioredoxin interacting protein
USP3: Ubiquitin-specific protease 3
VIM: Vimentin
Viperin: Virus inhibitory protein, endoplasmic reticulum associated, interferon inducible
VPS34: Vacuolar protein sorting 34
vRNA: Viral RNA
vRNP: Viral ribonucleoproteins
vtRNAs: Vault RNAs
ZIKV: Zika virus
